# Nitrogen deficiency identifies carbon metabolism pathways and root adaptation in maize

**DOI:** 10.1007/s12298-025-01631-0

**Published:** 2025-08-06

**Authors:** Joseph N. Amoah, Claudia Keitel, Brent N. Kaiser

**Affiliations:** School of Life and Environmental Sciences, University of Sydney, 380 Werombi Road, Brownlow Hill, Camden, NSW 2570 Australia

**Keywords:** Root system architecture, Nitrogen use efficiency, *Zea mays*, Root development, Adaptation

## Abstract

Sugars are essential for plant development, with nitrogen (N) availability playing a critical role in their distribution across plant organs, ultimately shaping growth patterns. However, the regulatory mechanisms modulating carbon (C) assimilate allocation and utilization under different N forms are not well understood. This study examined C fixation, utilization, and spatial re-distribution in the roots of hydroponically grown maize seedlings subjected to four N treatments: 1 mM NO_3_^−^ (low N; LN), 2 mM NO_3_^−^ (medium N; MN), 10 mM NO_3_^−^ (high N; HN), and 1 mM NH_4_^+^ (low ammonium; LA). LN treatment significantly increased soluble sugar, sucrose, and starch contents while promoting greater root biomass at the expense of shoot biomass, leading to a higher root to shoot assimilate allocation. The activities of sugar and starch metabolism enzymes were more tightly regulated under LN, indicating enhanced C utilization and increased competition for assimilates. Key genes involved in sugar (*ZmSPS*, *ZmSuSy*, *ZmSWEET6*, *ZmSUC2*, *ZmSTP2*, and *ZmAINV1*) and starch (*ZmAGPASE* and *ZmSS*) metabolism were upregulated under LN, correlating with increased root sucrose and starch accumulation and enhanced enzyme activity. Sucrose and starch accumulated predominantly in the brace and lateral roots. This pattern suggests that excess C accumulation results from inefficient C utilization in sink tissues rather than impaired C assimilation. These findings provide new insights into how LN modulates C partitioning in roots for stress adaptation, highlighting the importance of improving C utilization in sink tissues to mitigate N deficiency and enhance plant growth.

**Supplementary Information:**

The online version contains supplementary material available at 10.1007/s12298-025-01631-0.

## Introduction

 Nitrate (NO_3_^−^) and ammonium (NH_4_^+^) are integral macronutrients involved in various plant metabolic processes, including growth, development, and the production of harvestable yields. Carbon (C) is equally important to plant function, serving as both a structural component and primary energy source (Artins et al. 2024). C is captured and assimilated through photosynthesis and distributed across various organs to support metabolism and contribute to biomass accumulation (Cui et al. 2025). The intricate coordination between C and N metabolism is essential for plant productivity (Nunes Pires et al. 2024). N availability not only impacts photosynthetic efficiency and C assimilation, but also modulates C partioning among plant organs. Conversely, C supply to roots supports N uptake by promoting root growth and metabolic activity. This dynamic interplay between C and N modulates various developmental and physiological processes, playing an important role in plant adaptation, particularly under limited-N conditions (Zhao et al. 2020).

Plant roots serve as the primary organs for nutrient and water acquisition from the soil, facilitating plant growth and development. The availability and form of N significantly influence root system architecture, affecting both primary root (PR) and lateral root (LR) development (Sun et al. 2017; Kiba and Krapp 2016). For instance, previous studies have shown that N deficiency enhances PR and LR growth, whereas high N availability results in shorter PR and reduced LR numbers (Zhao et al. 2020; Xue et al. 2021). The plant root system consists of various components, such as LR, seminal roots (SR), PR, crown roots (CR), brace roots (BR), each playing a distinct role in N uptake to support plant growth and development (Zhang and Wu 2023). Root growth is rightly linked to the distribution of carbohydrates, especially under N deficient conditions. Source leaf-derived carbohydrates serve as intermediary substrates for metabolism, providing the necessary C for root growth and development (Wang and Ruan 2015; Zhao et al. 2020). Understanding the mechanisms influencing carbohydrate distribution in roots in response to different N forms is crucial for elucidating the interplay between C and N, as well as the adaptive responses of plant roots to varying soil N conditions.

Roots, as sink organs, require carbohydrates provided by leaves to grow and develop. Carbohydrate allocation depends on sink strength, which is related to the ability to unload and metabolize translocated carbohydrates (Prescott 2022). Sucrose is the predominant carbohydrate transported from source to sink organs in plants. This process relies on the efficient phloem transport system, which includes the symplastic and apoplastic pathways (Chen et al. 2017). Phloem loading or unloading of sucrose in roots involves a symplastic pathway through plasmodesmata, while the apoplastic pathway requires sucrose transporters, such as *SUTs*, *SWEETs*, and *SUCs* (Braun 2022). Following sucrose unloading in the apoplastic pathway, sucrose is converted into glucose and fructose by cell wall invertase and transported to root cells by SUTs on the plasma membrane. Sucrose unloading regulates sink strength and root cell growth. In sink cells, sucrose is converted to fructose and glucose by neutral invertase or to fructose and UDP-glucose by sucrose synthase (SuSy) activity. Sucrose is also transported to the vacuole, where it is converted to glucose and fructose by vacuolar acid invertase. In vacuoles, sucrose concentration regulates cell turgor and expansion, promoting root growth. N forms and levels can affect the activity and expression of enzymes involved in sugar metabolism, as shown in various plants (Ruan 2012; Zhang et al. 2021; Zhao et al. 2020; Huang et al. 2022). Previous studies have associated N deficiency with increased carbohydrate and nutrient ion allocation to roots (Terashima and Evans 1988), while N levels affect root sugar concentration (Dzamic and Stevanovic 1996). This finding has been confirmed in maize and citrus seedlings under varying NO_3_^−^ concentrations (Zhao et al. 2020; Huang et al. 2022). In contrast, NH_4_^+^ nutrition inhibited root development but promoted photosynthesis in Arabidopsis and wheat (Guo et al. 2007; Chen et al. 2020). However, there are few studies on sugar metabolism changes in roots under different N forms and levels. It remains unclear how root systems improve sugar metabolism efficiency to meet carbon requirements for growth under varying N forms.

Maize (*Zea mays* L.) was chosen for this study due to its significance as a staple food crop and its suitability as a model for investigating N metabolism and C allocation (Simons et al. 2014). Its growth patterns, responsiveness to environmental factors, and adaptability to various N conditions make it ideal for examining the effects of N forms and levels on carbon distribution (Dong et al. 2023). Optimizing N management in maize can significantly enhance global agricultural productivity (Begam et al. 2024). To elucidate the mechanisms underlying C allocation in different root systems, we examined the effects of various N forms on sucrose metabolism enzyme activities and transcript expression levels in maize seedlings. This study provides valuable insights into how different N forms influence C allocation and accumulation in the maize root system, aiding crop improvement strategies to address N deficiency or fluctuations. In this study, NO_3_^−^ levels were chosen based on Zhao et al (2020), while NH_4_^+^ concentration followed George et al (2016). Seedlings exposed to varying NO_3_^−^ levels, LN, MN, and HN, exhibited differential root growth, development and sugar accumulation. Zhao et al. (2020) showed that LN treatment promotes root growth and enhances C metabolism more effectively than MN and higher NO_3_^−^ levels. Conversely, small amounts of NH_4_^+^ were associated with improved root growth and photosynthesis in maize (George et al. 2016), whereas elevated NH_4_^+^ levels inhibited plant growth due to toxicity. High NH_4_^+^ concentrations can disrupt intracellular pH, osmotic balance, and nutrient absorption, leading to reduced growth and poor root development (Wang et al. 2022; Zhu et al. 2021). Therefore, using low NH_4_^+^ concentrations is essential for investigating C metabolism in maize root systems while avoiding the effects associated with higher levels of NH_4_^+^ nutrition.

This study aims to elucidate the impact of N deficiency on carbohydrate metabolism and root system architecture in maize, with a specific focus on how different N forms regulate sucrose utilization and partitioning, starch accumulation, and root plasticity. Through an integrative approach encompassing physiological, biochemical, and transcriptional analyses, the research seeks to unravel the mechanisms governing N-mediated C partitioning in maize roots. By evaluating these regulatory pathways, the findings will advance our understanding of nitrogen use efficiency (NUE), offering valuable insights into optimizing maize growth and resilience under N-deficient conditions. Ultimately, this research contributes to the development of sustainable agricultural practices and low input farming strategies, fostering improved crop productivity while minimizing environmental impact.

## Materials and methods

### Plant materials and seed treatment

Seeds of the fast-flowering, short-cycle inbred mini-maize line TX-40 J (McCaw et al. 2016), which were used in our previous experiment (Amoah and Kaiser 2025), were used in this study. Seeds were disinfected with 5% (v/v) sodium hypochlorite for 5 min and then washed with ultrapure MilliQ water, 5 times at 3 min each. Sterilized maize seeds were germinated in Oasis Horticube Propagation Slabs (Aqua Gardening, Australia), an inorganic and pH-neutral growing foam medium, placed in germination trays.

### Experimental treatment and growth conditions

The germination trays were transferred to a climate-controlled growth room, set to a 14/10 day-night cycle, with temperatures of 25 °C during the day and 22 °C at night, and 80% relative humidity for 5 d to allow for seed germination. After 5 days, the uniformly germinated seedlings were selected and divided into four treatment (T1-T4) groups and grown in 3L pots, which had the root ball supported by organic expanded clay pellets (Aqua Gardening, Australia). Each treatment group received specific N sources. Plants in T1 received 1 mM NO_3_^−^ (low N; LN), T2 received 2 mM NO_3_^−^ (medium N; MN), T3 had 10 mM NO_3_^−^ (High N; HN) and T4 received 1 mM NH_4_^+^ (low NH_4_^+^; LA). The NO_3_^−^ concentrations were chosen based on previous studies (Zhao et al. 2020), and the NH_4_^+^ concentration was selected based on the previous study (Peng et al. 2023). The hydroponic system was set up in a climate-controlled glasshouse, with conditions similar to those of the growth room, where the seeds were germinated but supplemented with LED lighting which provided 1000 µmol m^−2^ s^−1^ above pot level (Amoah and Kaiser 2025). Four systems were set up, and each system accommodated 40 pots, with each pot containing one plant. Plants were drip-irrigated with specified nutrient solutions (LN, MN, HN and LA), which were circulated through a hydroponic pump system. Irrigation occurred twice daily for 1 min, at 12:00 PM and 5:00 PM. Plants in each treatment groups were grown and treated using non-reticulated semi-hydroponic system conditions for 30 days before samples were harvested (Amoah and Kaiser 2025).

### Nutrient composition

The treatment solutions contained 1 mM (NH_4_)_2_SO_4_ (LA), 1 mM KNO_3_ (LN), 2 mM KNO_3_ (MN), or 10 mM KNO_3_ (HN), along with 1 mM, 2 mM, or 10 mM of MgSO_4_, KH_2_PO_4_, KCl, K_2_SO_4_, CaCl_2_, CaSO_4_, Fe-EDTA, Fe-EDDHA, H_3_BO_3_, MnSO_4_, ZnSO_4_, CuSO_4_, and Na_2_MoO_4_. Solutions were stored in 162 L Brute Containers with lids (Rubbermaid, USA) and were replaced weekly, with daily pH adjustments to maintain a stable pH of 5.9, using 1 M H_2_SO_4_ or 1 M NaOH. The treatment solution was delivered to the system via an Eden 140G FL submersible water pump (Creative Pumps, Australia). Plants were uniquely identified and randomized into blocks using the agricolae package R statistical software (v4.5.0).

### Photosynthesis and N content measurement

The net photosynthetic rate (Pn) was measured on the youngest expanded blade (YEB) of each treatment using the portable LI-6800 photosynthetic system (LI-COR Inc., Lincoln, NE, USA). Measurements were taken at 9:00 AM and 11:00 AM. Cuvette conditions included a light level of 1000 µmol m^−2^ s^−1^, CO_2_ concentration of 400 ppm, flow rate of 500 µmol m^−2^ s^−1^, and relative humidity between 60 and 65%. Total chlorophyll pigment was extracted from approximately 0.1 g of leaf tissue using 100% methanol on a shaker at 25 °C until the tissue was completely bleached. The extract was then centrifuged at 10,000 × g for 10 min, and the absorbance of the supernatant was measured at 652 and 663 nm using a spectrophotometer (UV-2550, Shimadzu, Japan). The concentration of chlorophyll was calculated following the method described by Amoah et al. (2023). N content was determined using a modified Kjeldahl method (Zhao et al. 2020). A 0.2 g dry sample was digested with 0.5 mL concentrated H_2_SO_4_ and a catalyst mixture (10 g K_2_SO_4_, 1 g CuSO_4_) at 100 °C for 60 min. After cooling, 0.5 mL of 40% NaOH and 0.5 mL of distilled water were added. The mixture was then combined with 1 mL Nessler’s reagent and incubated for 10 min. Absorbance at 420 nm was measured using a UV–Vis spectrophotometer (Shimadzu, Tokyo, Japan), and N content was calculated from a standard curve of (NH_4_)_2_SO_4_.

### Root measurements

At 30 DAT, uniformly grown maize seedlings were sampled and divided into two sets (Set I and Set II), each comprising 12 individual plants per treatment group (T1–T4). In Set I, whole roots were carefully separated from shoots, rinsed with potable water, and processed as follows: roots from six plants were floated in water in a transparent plastic tray and scanned using an Epson Perfection V700 photo scanner (Epson Australia Pty. Ltd., Australia) for morphological analysis. Subsequently, the roots were blotted with tissue paper and oven-dried at 70 °C for 48 h to determine total root biomass. The remaining six plants were immediately frozen in liquid nitrogen and stored at − 80 °C for subsequent biochemical and molecular analyses. In Set II, roots were dissected into distinct types: brace roots (BR), crown roots (CR), lateral roots (LR), primary roots (PR), and seminal roots (SR). Roots from six plants were scanned as described above, then oven-dried at 70 °C for 48 h to assess individual root-type biomass. The remaining six plants were used for biochemical and molecular analyses, with each root type frozen separately in liquid nitrogen and stored at − 80 °C. Image analysis was performed using RhizoVision Explorer software (version 2.0.3) (Seethepalli et al. 2021).

### Quantification of soluble sugar and starch

Soluble sugar and sucrose contents were determined following the method described by Xiao et al. (2024). Ground samples (0.1 g) were homogenized in 1 mL of 80% (v/v) ethanol and heated at 80 °C for 30 min. After cooling for 5 min, the mixture was centrifuged at 12,000 × g for 10 min. The supernatants were collected to determine soluble sugar and sucrose contents using a UV–Vis spectrophotometer (Shimadzu, Tokyo, Japan), with absorbance recorded at 620 nm and 480 nm, respectively. For starch content, 1 mL of distilled water was added to the ethanol-insoluble residues and incubated for 30 min. Starch was hydrolyzed with 9.2 M and 4.6 M perchloric acid solutions. The starch content was quantified using anthrone reagent, and absorbance was measured at 620 nm (Du et al. 2020).

### Starch metabolism enzymes activity assay

For starch synthase (SS) activity, 100 mg of tissue samples were homogenized in an extraction buffer containing 50 mM Tris–HCl (pH 7.0), 10% glycerol, 10 mM EDTA, 5 mM DTT, 1 mM PMSF, and 50 μL/g tissue of 10 × Protease Inhibitor Cocktail (Sigma-Aldrich, Cat# P9599) (Cao et al. 1999). The homogenate was centrifuged at 12,000 × g for 10 min, and the supernatant was collected. A reaction mixture was prepared by mixing 0.1 mL of the supernatant with 0.9 mL of a solution containing 50 mM Tris–HCl (pH 7.0), 5 mM ADP-glucose, 1 mg/mL glycogen, and 10 mM MgCl_2_. The reaction mixture was incubated at 30 °C for 30 min. To stop the reaction, 0.1 M HCl was added to denature the enzymes. To detect inorganic phosphate (Pi) consumption, 1% (w/v) ammonium molybdate was added. The mixture was incubated at room temperature for 30 min, and the absorbance was recorded at a wavelength of 620 nm using a UV–Vis spectrophotometer (Shimadzu, Tokyo, Japan). A standard curve was prepared using known Pi concentrations, and starch synthase activity was calculated as the amount of Pi released, expressed in μmolg^−1^FW.

ADP-glucose pyrophosphorylase (AGPase) activity was determined using previously described methods by Prathap et al. (2019), with minor modifications. Briefly, 100 mg of fresh samples were homogenized in 1 mL of ice-cold extraction buffer containing 0.1 M Tris–HCl (pH 7.9), 5 mM glutathione, and 1 mM EDTA. The homogenate was centrifuged at 15,000 × g for 20 min at 4 °C, and the supernatant was collected. Subsequently, 0.1 mL of the supernatant was mixed with 0.9 mL of a reaction mixture containing 0.4 M Tris–HCl buffer (pH 7.9), 0.06 M MgSO_4_, 48 mM cysteine, 2.4 mg/mL BSA, 4 mM ADP-glucose, 20 mM sodium pyrophosphate, 30 mM 3-phosphoglycerate, and 4 units each of glucose-6-phosphate dehydrogenase and phosphoglucomutase. Afterwards, 0.1 mL of enzyme extract was added to NADP^+^ as the final component. The absorbance was measured at 340 nm using a UV–Vis spectrophotometer (Shimadzu, Tokyo, Japan). The AGPase activity was expressed as μmol min^−1^ g^−1^ FW.

To determine SPS and SuSy activity, a 0.1 g of frozen tissue samples were homogenized in an extraction buffer containing 50 mM Tris–HCl (pH 7.5), 1 mM EDTA, 1 mM MgCl_2_, 12.5% (v/v) glycerine, 10% polyvinylpyrrolidone (PVP), and 10 mM mercaptoethanol to ascertain the activities of sucrose metabolism-related enzymes. The SPS and SuSy activities were measured using the extract (Liu et al. 2013). Briefly, 200 μL of supernatant was mixed with reaction buffer containing 200 mM Tris–HCl (pH 7.0), 40 mM MgCl_2_, 12 mM UDP-glucose, 40 mM fructose-6-P, and 200 μL extract. Another reaction buffer containing 12 mM UDP, 40 mM sucrose, 200 mM Tris–HCl (pH 7.0), and 40 mM MgCl_2_ was also prepared. The mixture was incubated at 30 °C for 30 min and terminated using 100 μL 2 mol L^−1^ of NaOH. The mixture was then heated at 100 °C for 10 min to destroy untreated hexose and hexose phosphates, cooled to room temperature, and mixed with 1 mL of 0.1% (w/v) resorcin in 95% (v/v) ethanol and 3.5 mL of 30% (w/v) HCl. The solution was incubated for 10 min at 80 °C. Sucrose content in the SPS reaction and fructose content in the SuSy reaction were calculated using a standard curve measured at A480 nm and A540 nm wavelengths, respectively.

### RNA isolation, cDNA synthesis, and qPCR analysis

Total RNA was isolated from the whole root and the different root types (BR, CR, LR, PR and SR) using the TRIzol RNA Isolation Reagents ( Invitrogen, Carlsbad, CA, USA) following the manufacturer’s protocol. RNA quantity and integrity were assessed by measuring the optical density at 260 nm and through 1.0% (w/v) agarose gel electrophoresis, respectively. Subsequently, 1 µg of total RNA was reverse-transcribed into single-stranded cDNA using the iScript™ RT Reagent Kit (Bio-Rad, Hercules, CA, USA) according to the manufacturer’s instructions. Quantitative real-time polymerase chain reaction (qPCR) was performed using the CFX 96 Real-Time System (Bio-Rad, Richmond, CA, USA) with SYBR Green fluorescence (Bio-Rad, Richmond, CA, USA). The ∆∆CT method was used for data analysis. Gene-specific primers (Table S1) were employed to assess their expression patterns under different N (LN, MN, HN and LA) treatment conditions. The thermal cycling conditions consisted of an initial denaturation step at 95 °C for 5 min, followed by 40 cycles of 95 °C for 15 s, 55 °C for 15 s, and 72 °C for 30 s. All experiments were conducted with three biological replicates, and relative transcript levels were normalized using *ZmActin* and *ZmUBQ* as internal controls.

### Statistical analysis

The study was repeated twice, with tissue samples collected in triplicate at 30 DAT. Data were analysed using a one-way analysis of variance (ANOVA) to assess overall differences among groups, followed by post-hoc Turkey’s honest significant difference (HSD) test for pairwise comparison using Prism software (v10.0). Quantified data points represent the mean ± standard error (SE) of six independent plants (n = 6). Different letters on the error bars denote statistically significant differences at a probability level of *p* ≤ 0.05, determined via Tukey’s HSD test. Graphical charts were generated using GraphPad Prism (v10.4.0), and Pearson’s correlation plots were created using R statistical software (v4.5.0).

## Results

### Phenotypic response, biomass, and photosynthesis under different N forms

At 30 DAT, maize plants grown under HN conditions exhibited enhanced shoot growth, whereas LN-treated plants showed increased root growth (Figs. S1A–B). Consistent with these phenotypic changes, LN treatment significantly (*P* ≤ 0.05) inhibited shoot biomass while promoting root biomass accumulation, leading to a higher total biomass and an increased root-to-shoot (R/S) ratio in maize seedlings (Figs. S1A-C and S3A). Furthermore, LN treatment was associated with reduced N concentration, Pn, and chlorophyll content in both the leaves and roots of maize seedlings (Figs. S2D and S2B, S3C–D)). Additionally, N treatments (NT) significantly (*P* ≤ 0.05) affected root and shoot biomass, N content, and the R/S ratio (Table S2).

### Changes in root morphology under different N treatment forms

The morphological changes associated with LN, MN, HN, and LA treatment conditions were examined after 30 days. As shown in Fig. S2, LN-treated plants exhibited significantly increased total root length, a higher number of root tips, greater root volume, and an expanded total root surface area (Fig. S2E–H). These traits corresponded with a significant increase in root biomass compared to other treatment groups (Fig. S2B). All measured root morphological traits were significantly (*P* ≤ 0.05) affected by the different nitrogen treatment (Table S2).

### Soluble sugars and sucrose metabolism enzymes activities under different N forms

**Fig. 1:**
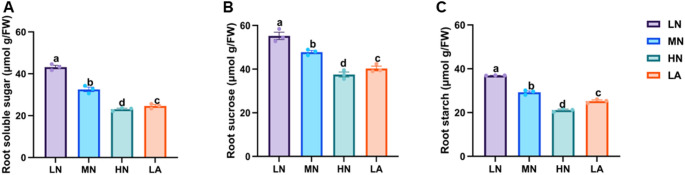
Effects of different nitrogen (N) treatments on soluble sugar (**A**), sucrose (**B**), and starch (**C**) contents in the whole roots of maize inbred line TX-40 J. Data represent mean ± standard error (SE) of six plants (n = 6). Different letters on error bars indicate statistically significant differences at *P* ≤ 0.05. FW fresh weight, LN low nitrate, MN moderate nitrate, HN high nitrate and LA low ammonium

**Fig. 2:**
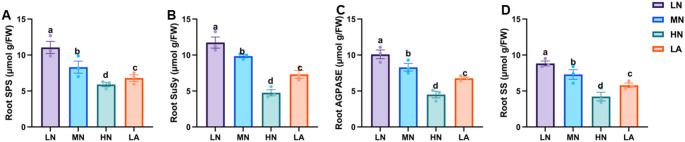
Activities of sucrose phosphate synthase (**A**), sucrose synthase (**B**), ADP-glucose pyrophosphorylase (**C**), and starch synthase (**D**) in the whole roots of maize seedlings under different nitrogen (N) treatments. Data represent mean ± standard error of the mean (SEM) of six plants (n = 6). Statistical significance was assessed using Tukey’s multiple range test (*P* < 0.05); different letters indicate significant differences between treatments. FW fresh weight, LN low nitrate, MN moderate nitrate, HN high nitrate and LA low ammonium

LN treatment significantly (*P* ≤ 0.05) increased total soluble sugar, sucrose, and starch contents in the roots of maize seedlings compared to other nitrogen-treated plants (Fig. 1A–C). Similarly, the activities of sugar metabolism enzymes (SPS and SuSy) and starch metabolism enzymes (AGPASE and SS) were markedly (*P* ≤ 0.05) higher in the roots of LN-treated plants than in those under MN, HN, and LA treatments (Fig. 2A–D). Additionally, soluble sugar, sucrose, starch, and the activities of SPS, SuSy, AGPASE, and SS were all significantly (*P* ≤ 0.05) influenced by NT (Table S2).

### Transcriptional regulation of sucrose-metabolism genes under different N forms

**Fig. 3:**
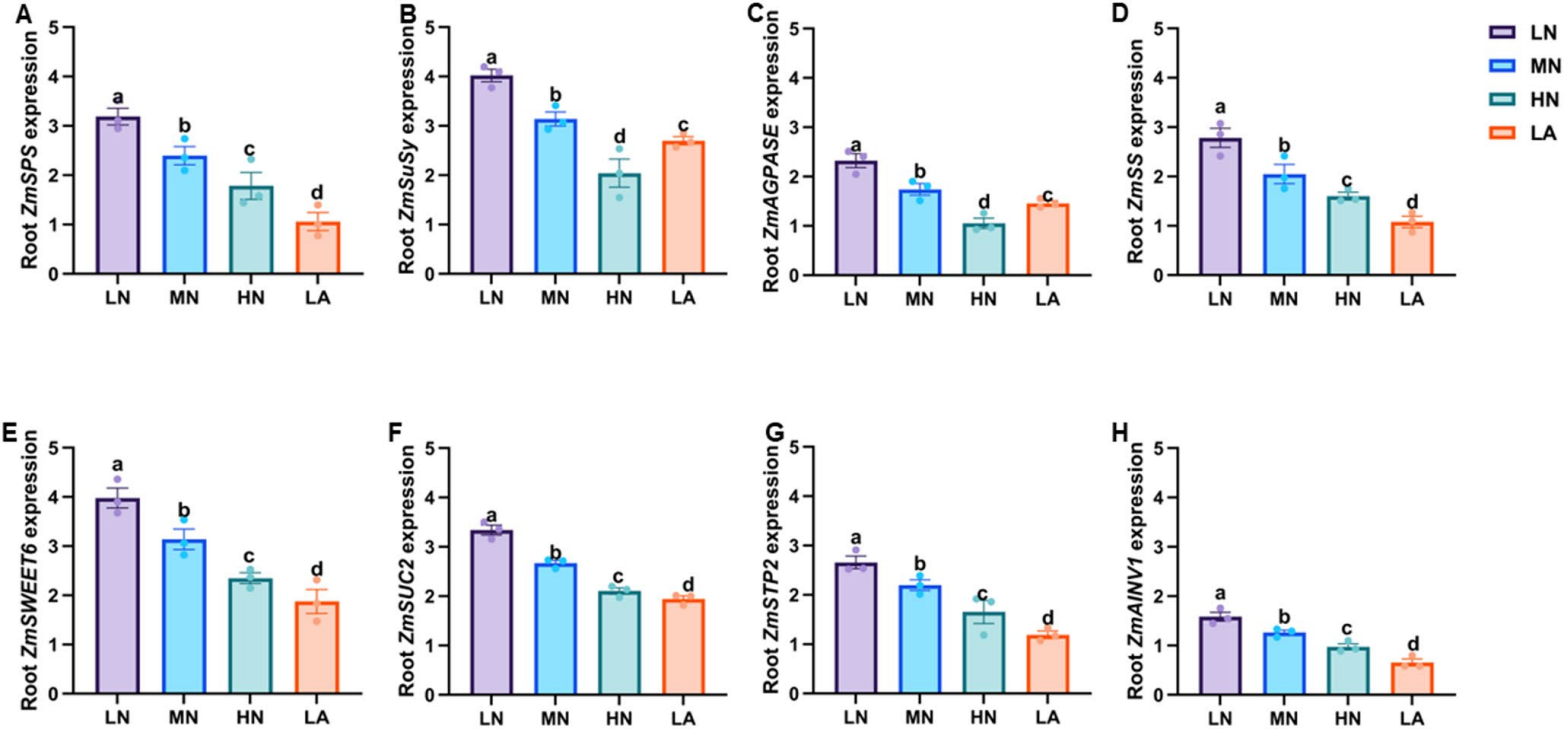
Effects of different nitrogen (N) treatments on the expression patterns of sugar and starch metabolism-related genes in the whole roots of maize inbred line TX-40 J. Expression levels of *ZmSPS* (**A**), *ZmSuSy* (**B**), *ZmAGPASE* (**C**), *ZmSS* (**D**), *ZmSWEET6* (**E**), *ZmSUC2* (F), *ZmSTP2* (**G**), and *ZmAINV1* (**H**). Data represent mean ± standard error of the mean (SEM) of six plants (n = 6). Statistical significance was determined by Tukey’s multiple range test (*P* < 0.05); different letters indicate significant differences among treatments. LN low nitrate, MN moderate nitrate, HN high nitrate and LA low ammonium

The expression patterns of sucrose metabolism and transporter-related genes (*ZmSPS*, *ZmSuSy*, *ZmAINV1* and *ZmSUC2*) were analysed in the roots of maize plants under LN, MN, HN, and LA treatment conditions. As shown in Fig. 3, LN treatment significantly (*P* ≤ 0.05) upregulated the expression of *ZmSPS*, *ZmSuSy*, *ZmSWEET6*, *ZmSUC2*, and *ZmAINV1*), with greater fold changes compared to other treatment groups (Fig. 3A, B, E, F, G and H). Similarly, the expression of starch metabolism genes (*ZmAGPASE* and *ZmSS*) was also markedly upregulated in the roots of LN-treated plants compared to those in other treatment groups (Fig. 3C–D). Interestingly, the type of nitrogen treatment significantly influenced the expression of all genes analysed (Table S2).

### Diurnal changes of sugars and starch under different forms

**Fig. 4:**
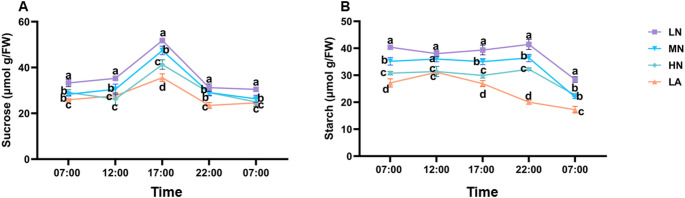
Impact of different nitrogen (N) forms on the diurnal patterns of sucrose (**A**) and starch (**B**) contents in the whole roots of maize seedlings. Samples were collected at 7:00, 12:00, 17:00, 22:00, and 7:00 on the following day. Data represent mean ± standard error of the mean (SEM) of six plants (n = 6). Statistical significance was determined by Tukey’s multiple range test (*P* < 0.05); different letters indicate significant differences among treatments. FW fresh weight, LN low nitrate (N deficiency), MN moderate nitrate, HN high nitrate and LA low ammonium

Plants subjected to LN treatment consistently exhibited increased sucrose and starch levels throughout the day. These sucrose and starch levels were significantly different (*P* ≤ 0.05) from those observed in other treatment groups (Fig. 4A–B). The diurnal pattern of sucrose level showed the lowest values in the early morning (7:00 AM), followed by an increase at 12:00 PM, peaking at 5:00 PM, and declining overnight to reach their lowest levels again at 7:00 AM the next day. The diurnal starch level was also lower in the early morning, increased at 12:00 PM and peaked at 22:00 PM and reached the lowest again at 7:00 AM the next day. Furthermore, after overnight transport, plants under LN treatment retained significantly (*P* ≤ 0.05) higher root sucrose and starch concentrations than those in other treatment groups (Fig. 4A–B).

### Biomass and morphology of different root types under different N condition

To evaluate the effects of LN, MN, HN, and LA treatments, whole roots were categorized into five primary types: BR, CR, PR, LR and SR. As shown in Fig. S4, HN treatment significantly promoted the biomass accumulation of BR, CR, PR, and SR in maize seedlings. However, LR growth was strongly influenced by LA treatment (Fig. 6C). The magnitude of biomass accumulation was highest in CR, LR, and SR, but lower in BR and PR. In contrast, the LN treatment revealed inhibited BR, CR, PR, and SR growth. Furthermore, the LN treatment significantly (*P* ≤ 0.05) increased the length of all root types (Figs. S5A–D), enhanced LR tip number (Figs. S5E–F), and increased the volume of CR, LR, and PR (Figs. S6A–D), as well as the surface area of CR, PR, and LR (Figs. S6E–F). MN treatment stimulated CR and LR tip number and increased PR volume and LR surface area (Figs. S5 and S6). However, HN treatment concurrently significantly inhibited the morphological development of various root types, leading to reduced total root length, number of root tips, root volume and root surface area. The nitrogen treatment significantly impacted CR, LR, PR, and SR growth in maize seedlings (Table S2).

### Soluble sugars and sucrose metabolism enzyme activities in different root types

**Fig. 5:**
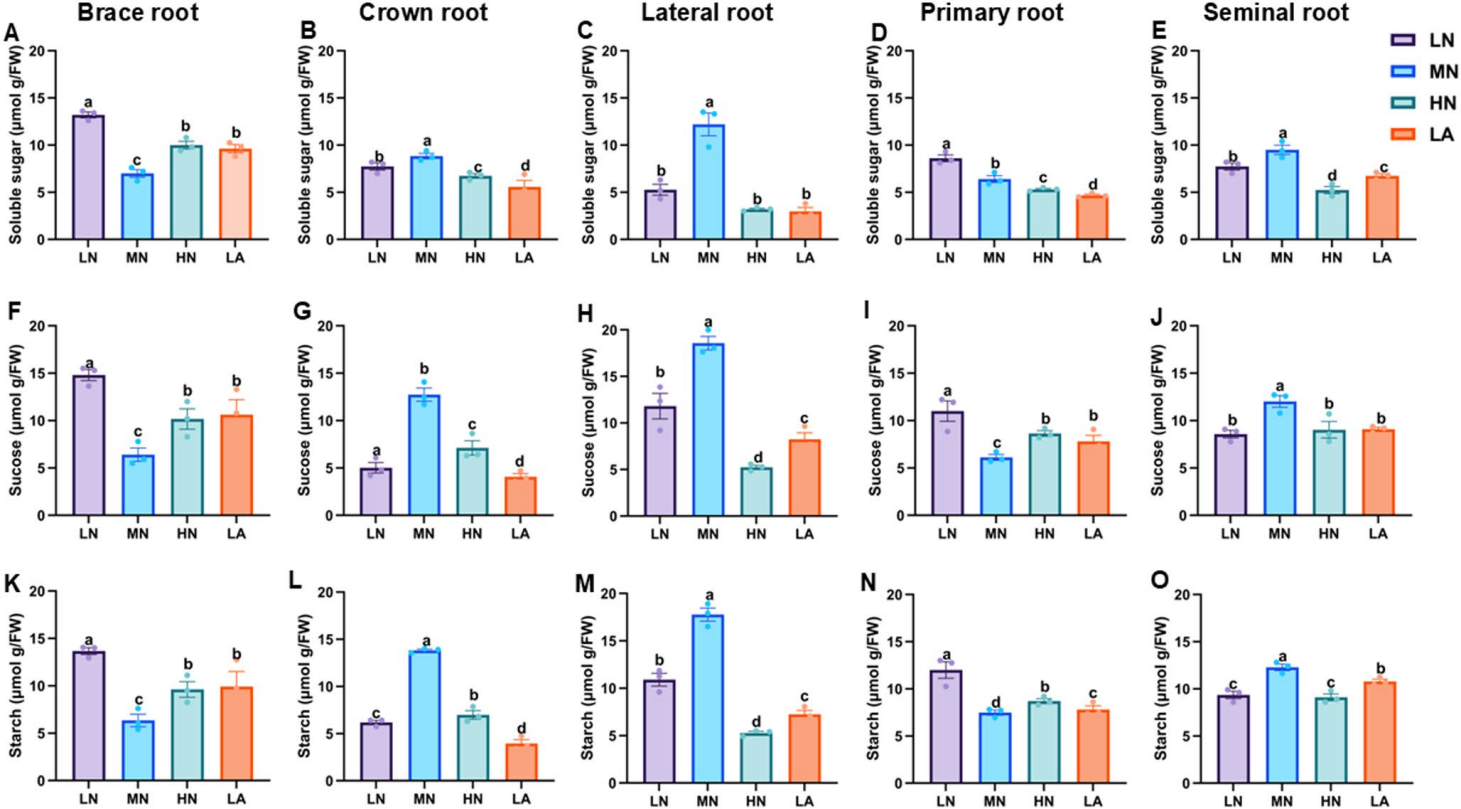
Effect of different nitrogen (N) forms on sugars and starch content in different root types of maize inbred line TX-40 J. Soluble sugar content in brace (**A**), crown (**B**), lateral (**C**), primary (**D**) and seminal root (**E**). Sucrose content in brace (**F**), crown (**G**), lateral (**H**), primary (**I**) and seminal root (**J**), and starch content in brace (**K**), crown (**L**), lateral (**M**), primary (**O**) and seminal root (**P**). Data represent mean ± standard error of the mean (SEM) of six plants (n = 6). Statistical significance was determined by Tukey’s multiple range test (*P* < 0.05); different letters indicate significant differences among treatments. LN low nitrate, MN moderate nitrate, HN high nitrate and LA low ammonium

LN treatment significantly (*P* ≤ 0.05) increased soluble sugar, sucrose, and starch contents in BR and PR (Fig. 5). In contrast, MN treatment enhanced sugar, sucrose, and starch accumulation in the CR, LR and SR (Figs. S5). Consistent with these changes in sugar and starch content, LN treatment significantly increased the activities of sucrose and starch metabolism enzymes (SPS, SuSy, AGPASE, and SS) in BR and SR, whereas MN treatment enhanced the activities of these enzymes in CR, LR, and PR, respectively (Table S3). Additionally, soluble sugar, sucrose, starch content, and the activities of SPS, SuSy, AGPASE, and SS were all significantly affected by N T (Table S2).

### Expression of sucrose and starch metabolism-related genes in different root types

**Fig. 6:**
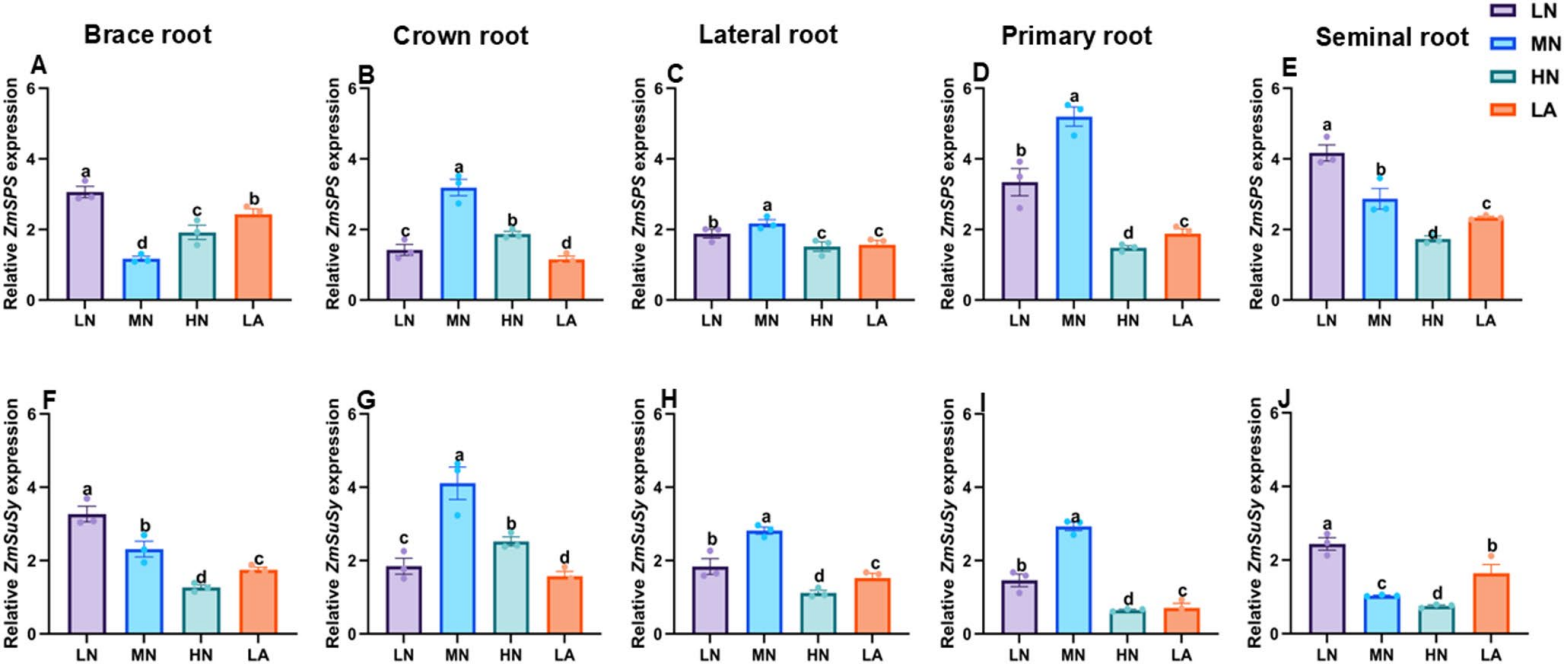
Effects of different nitrogen (N) treatments on the expression patterns of sugar metabolism-related genes in different root types of maize inbred line TX-40 J. Expression levels of *ZmSPS* in brace, crown, lateral, primary, and seminal roots (**A**–**E**), and *ZmSuSy* in brace, crown, lateral, primary, and seminal roots (**F**–**J**) are shown. Data represent mean ± standard error of the mean (SEM) of six plants (n = 6). Statistical significance was determined by Tukey’s multiple range test (*P* < 0.05); different letters indicate significant differences among treatments. LN low nitrate, MN moderate nitrate, HN high nitrate and LA low ammonium

**Fig. 7:**
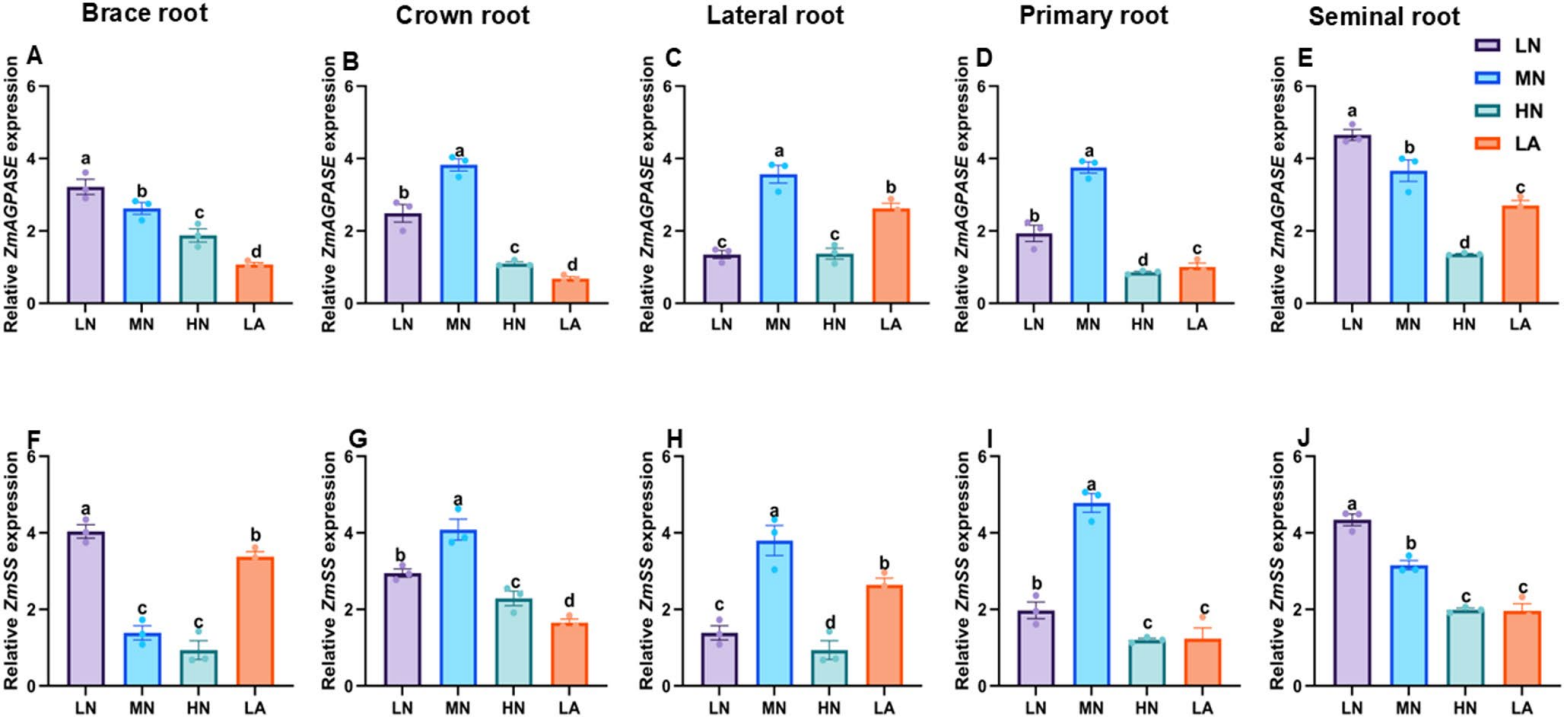
Effects of different nitrogen (N) treatments on the expression patterns of starch metabolism-related genes in different root types of maize inbred line TX-40 J. Expression levels of *ZmAGPASE* in brace, crown, lateral, primary, and seminal roots (**A**–**E**), and *ZmSS* in brace, crown, lateral, primary, and seminal roots (**F**–**J**) are shown. Data represent mean ± standard error of the mean (SEM) of six plants (n = 6). Statistical significance was determined by Tukey’s multiple range test (*P* < 0.05); different letters indicate significant differences among treatments. LN low nitrate, MN moderate nitrate, HN high nitrate and LA low ammonium

**Fig. 8:**
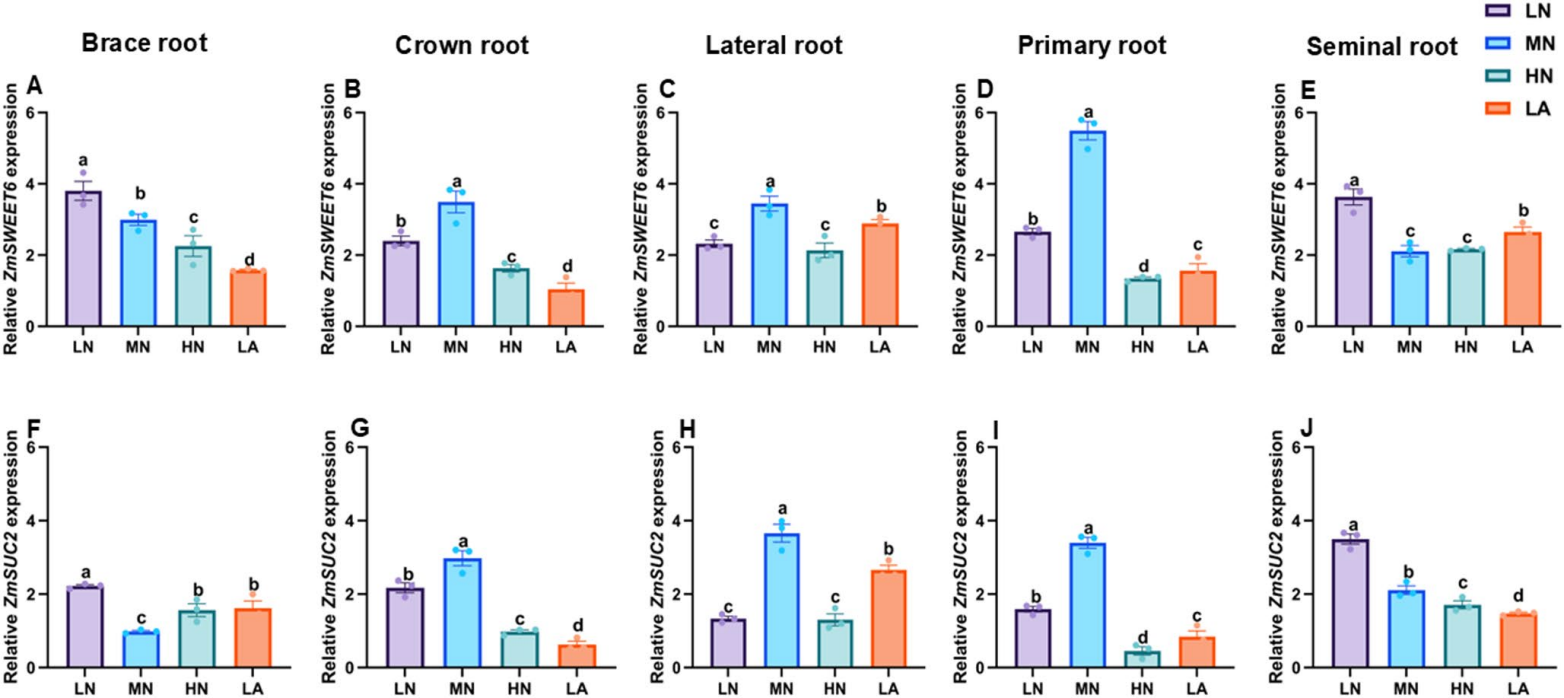
Effects of different nitrogen (N) treatments on the expression patterns of sucrose metabolism-related genes in different root types of maize inbred line TX-40 J. Expression levels of *ZmSWEET6* in brace, crown, lateral, primary, and seminal roots (**A**–**E**), and *ZmSUC2* in brace, crown, lateral, primary, and seminal roots (**F**–**J**) are shown. Data represent mean ± standard error of the mean (SEM) of six plants (n = 6). Statistical significance was determined by Tukey’s multiple range test (*P* < 0.05); different letters indicate significant differences among treatments. LN low nitrate, MN moderate nitrate, HN high nitrate and LA low ammonium

**Fig. 9:**
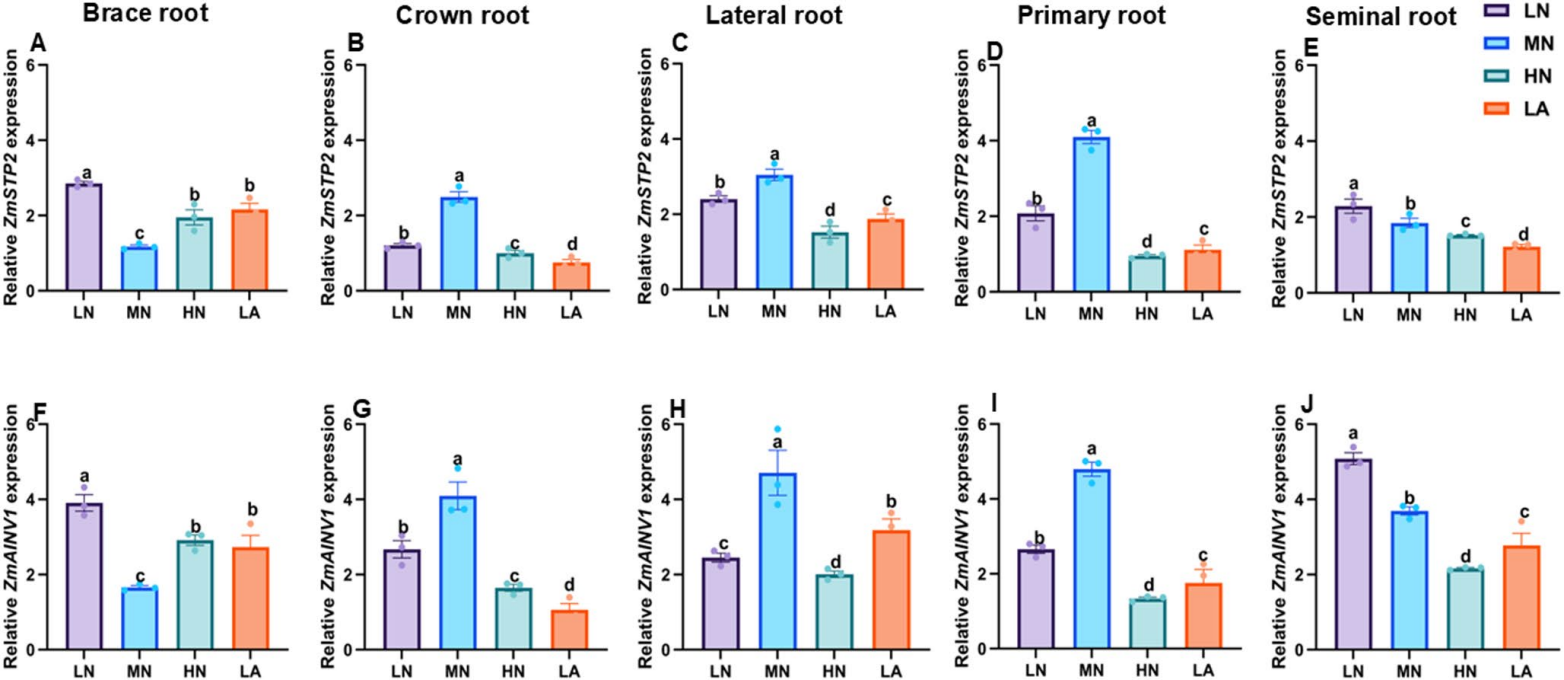
Effects of different nitrogen (N) treatments on the expression patterns of sucrose transporter (*ZmSTP2*) and sugar metabolism (*ZmAINV1*) genes in different root types of maize inbred line TX-40 J. Expression levels of *ZmSTP2* in brace, crown, lateral, primary, and seminal roots (**A**–**E**), and *ZmAINV1* in brace, crown, lateral, primary, and seminal roots (**F**–**J**) are shown. Data represent mean ± standard error of the mean (SEM) of six plants (n = 6). Statistical significance was determined by Tukey’s multiple range test (*P* < 0.05); different letters indicate significant differences among treatments. LN low nitrate, MN moderate nitrate, HN high nitrate and LA low ammonium

LN treatment significantly (*P* ≤ 0.05) upregulated the expression of *ZmSPS*, *ZmSuSy, ZmAGPASE*, *ZmSS*, *ZmSWEET6*, *ZmSUC2*, *ZmSTP2*, and *ZmAINV1* in BR (Figs. 6, 7, 8, 9A and F) and SR (Figs. 6, 7, 8, 9E–F). In contrast, the expression of these genes was highly upregulated in CR (Figs. 6, 7, 8, 9B and G), as well as LR and PR (Figs. 6, 7, 8, 9D and I) in plants under MN treatment. Additionally, NT significantly affected the expression levels of *ZmSPS, ZmSuSy*, *ZmAGPASE*, *ZmSS*, *ZmSWEET6*, *ZmSUC2*, *ZmSTP2*, and *ZmAINV1* (Table S2).

## Discussion

Nitrogen (N) is an essential nutrient that shapes biomass distribution between shoots and roots. Its various forms and concentrations elicit distinct plant responses, with LN and NH_4_^+^ supply often inhibiting shoot growth while promoting root development (Sun et al. 2021). While extensive studies have examined N responses in different crop species, research in maize has primarily remained limited in focus to single N form, leaving gaps in the understanding how different N forms influences growth and C metabolism. Evaluating the impact of various N forms is essential for elucidating plant adaptation mechanisms. Studies indicate that NO_3_− and NH_4_^+^, differentially affect maize root architecture and nutrient uptake efficiency (The et al. 2021), while fluctuations in N availability influences C partitioning, starch accumulation and sucrose metabolism (Zhang et al. 2020).

### Physiological adaptations to different nitrogen forms in maize roots

In this study, the LN treatment inhibited aerial growth, as evidenced by a reduction in shoot biomass, lower Pn, and decreased chlorophyll and total N content in both the leaves and roots of maize seedlings (Figs. S1A, S2A–D and S3A–D). These findings align with previous studies demonstrating that N deficiency impairs growth and photosynthetic efficiency, limiting C assimilation and shoot expansion (Zhao et al. 2020). Conversely, LN treatment stimulated overall root growth at the whole root system level, increasing the R/S ratio and enhancing total biomass accumulation (Figs. S2B–C). The increased overall root development expands the root system, improving the plant’s ability to absorb N from the soil under N-deficient conditions (Kiba and Krapp 2016). A higher R/S ratio indicates that maize seedlings prioritize C allocation to the roots, strengthening resilience to N stress and supporting overall growth (Lopez et al. 2023). Despite the inhibition of shoot growth, the increase in total biomass in LN-treated plants highlights their compensatory response, expanding root structures to enhance root development and N acquisition (Zhao et al. 2020). At the whole root level, maize plants grown under LN treatment exhibited greater root length, higher root number, increased root volume, and expanded root surface area (Fig. S2E–H). These overall root architectural modifications enhance N uptake efficiency, enabling LN-treated plants to better adapt and survive under N deficient conditions, consistent with previous findings (Sun et al. 2021). Furthermore, these results emphasize the critical role of root plasticity in maintaining plant growth and development under suboptimal N availability (Awasthi and Laxmi 2021). The findings also highlight the importance of root system modulation as a strategic adaptation to N deficiency, providing valuable insights into resource allocation optimization for improved plant resilience and productivity.

Moreover, examining the distinct physiological and architectural traits of different maize root types offers valuable insight into the mechanisms driving the overall whole-root response (Protto et al. 2024). While the whole root system showed overall promotion of growth and root architecture, the mechanism underlying assimilate allocation in different root types under various N forms shows distinct growth patterns and developmental responses across specific root types in response to varying N treatments. LN treatment inhibited the growth of BR, CR, SR and PR, while promoting LR development (Figs. S5 and S6). This demonstrates a strategic shift in root architecture under N-limited conditions. The decreased biomass of BR, CR, PR, and SR under LN suggests that LN plants prioritize assimilate allocation away from these root types, which may be due to their high metabolic cost and lower efficiency in N uptake under N-limited conditions (Khan et al. 2019). Conversely, the enhanced LR proliferation under LN treatment represents an adaptive response to increase surface area for nutrient absorption (Pélissier et al. 2021). Contributing to the overall increase in whole-root length, plants grown under LN treatment exhibited increased root length across all root types (Figs. S5A–D). This was supported by a higher number of LR tips and expanded CR, PR, and LR volume and surface area. These specific morphological adaptations in root types, particularly the extensive development of lateral roots and expansion of surface area (Figs. S5 and S6), would contributed significantly to enhanced N acquisition capacity observed at the whole root level under conditions of LN condition (Giehl et al. 2014).

Furthermore, as a signalling molecule, NO_3_− has been shown to regulate root system architecture (RSA) (Asim et al. 2020). In this study, LN treatment promoted LR elongation, improving their ability to explore a larger soil volume for N uptake (Figs. S5 and S6). This observation is consistent with previous findings (Gruber et al. 2013), suggesting that maize seedlings actively remodel their RSA to compensate for LN supply. The expansion of CR, PR, and LR under LN conditions demonstrates a coordinated developmental response aimed at increasing the absorptive interface between the root system and the soil matrix (López-Bucio et al. 2003). This dynamic shift toward a more extensively branched root network may contribute to improved NUE and enhanced adaptation to N-deficient conditions observed at the whole plant level (Figs. S2E–H).

### Effects of different N forms on C–N coordination in maize roots and specific root types

N availability plays a crucial role in root formation, and this process is closely linked to C metabolism (Zhao et al. 2020). The observed physiological and architectural adaptations under N deficiency are underpinned by coordinated biochemical and molecular changes in C metabolism throughout the root system, with distinct patterns across different root types (Fig. 5). At the whole root level, N form led to differential sugar and starch accumulation in maize seedlings. While LN plants consistently accumulated higher levels of soluble sugar and sucrose (Figs. 1A,B), indicating a strong allocation of photosynthetic assimilates to roots under N deficiency (The et al. 2021). The LA treatment also influenced C metabolism, although with a distinct pattern (Fig. 1). The activities of SPS and SuSy were significantly elevated at the whole root level (Fig. 2A,B), alongside the upregulated expression of *ZmSPS*, *ZmSuSy*, *ZmAINV1*, *ZmSWEET6*, *ZmSUC2*, and *ZmSTP2* in the LA-treated whole roots (Fig. 3A,B,E–H). The enhanced accumulation of carbohydrates in roots may be attributed to an increased capacity for unloading photosynthetic assimilates, thereby facilitating root growth and adaptation to N deficiency. These findings emphasize the critical role of N availability in regulating C partitioning and root development, which is vital for improving NUE (Amoah and Kaiser 2025).

Sucrose loading in the phloem at the source and its unloading at the sink creates a turgor pressure difference, driving the mass flow of assimilates across tissue boundaries (Lalonde et al. 2003). To maintain this pressure gradient and ensure continuous sucrose unloading into the roots, sucrose must be degraded (Ruan 2014). Under LN treatment, enhanced maize root growth and development depended on sucrose as an energy source, with increased SuSy activity alongside upregulated expression of *ZmSPS*, *ZmSuSy* and *ZmAINV1* (Fig. 3) as seen in other studies (Zhao et al. 2020). Given the role of SuSy as a marker of sink strength (Zhao et al. 2020), its elevated activity and expression suggest a key function in enhancing whole-root sink strength under LN conditions. *STPs* facilitate sucrose transport from source tissues, promoting root growth and development (Wu et al. 2024a, b). In this study, *ZmSTP2* expression was significantly higher at the whole-root level under LN, indicating its strong induction in response to N deficiency and its role in efficient sucrose transport to the roots, ensuring an adequate energy supply for growth. *SUCs* contribute to sucrose breakdown into glucose and fructose, providing energy and C skeletons for various metabolic processes (Ruan 2012). This expression is often upregulated under N deficiency, accelerating sucrose degradation to support root cell division and expansion (Wang et al. 2021). Here, *ZmSUC2* was upregulated at whole-root level under LN treatment (Fig. 3), reinforcing its role in sucrose degradation and energy supply for root development in N-limited conditions (Zhu et al. 2022).

LN treatment increased root starch content, indicating a shift in C metabolism under N deficiency (Fig. 1C). Starch a crucial energy and C storage compound, is strongly influenced by N availability (Amoah and Kaiser 2025). The increased whole-root starch content observed in LN-treated plants suggests enhanced C storage, demonstrating a strategy where LN plants prioritize starch accumulation in roots when shoot growth is inhibited due to N limitation (Zhao et al. 2020). Under N deficient conditions, reduced shoot growth may lower assimilate demand, consequently decreasing carbohydrate allocation to developing tissues and leading to starch accumulation in the roots. This buildup may be attributed to reduced sink strength (Aluko et al. 2023). Furthermore, N deficiency can impose metabolic constraints, particularly on enzymatic activities involved in C partitioning, thereby affecting starch metabolism and utilization efficiency in plants (Liu et al. 2025). Thus, starch accumulation under LN conditions may not solely indicate improved stress adaptation but rather reflect reduced sink strength or metabolic constraints. Consistent with whole-root starch accumulation, LN significantly increased AGPASE and SS activities while upregulated the expression of *ZmAGPASE* and *ZmSS* at the whole-root level (Figs. 3C–D, 4C–D, and S3). The Increased AGPASE and SS activities and gene-upregulation *ZmAGPASE* and *ZmSS* at the whole-root highlight enhanced starch synthesis, ensuring a stable energy supply for root expansion and adaptation under N-deficient conditions, as previously demonstrated (Li et al. 2018).

The differential effects of N treatments on soluble sugar, sucrose, and starch accumulation across maize root types illustrate dynamic C partitioning in response to N availability (Zhao et al. 2020). Under LN, sugar and starch contents increased specifically in BR and PR, suggesting a strategic allocation of C reserves to enhance deep-rooted structural stability and nutrient acquisition (Fig. 5). This aligns with previous findings demonstrating that N availability modulates carbohydrate metabolism to optimize root function (Khan et al. 2019; Wang et al. 2021). Conversely, MN treatment led to higher sugar and starch accumulation in CR, LR, and SR, directing C toward root types associated with increased proliferation and exploration under MN availability (Yu et al. 2016). Additionally, SPS, SuSy, AGPASE, and SS activities correlated with these changes in sugar accumulation, reinforcing the role of N in regulating carbohydrate metabolism to optimize specific root-type development (Fig. S8). Under LN conditions, elevated enzymatic activity in BR and SR likely enhances C utilization in structural and absorptive root segments (Table S3). Meanwhile, increased enzyme activity in CR, LR, and PR under MN suggests a metabolic adaptation favouring LR expansion and N acquisition in those conditions (Lemoine et al. 2013). These findings underscore the intricate interplay between N availability and C partitioning across maize root types, shaping the overall C status observed at the whole-root level (Fig. 1). At the molecular level, LN upregulated genes involved in sucrose transport (*ZmSWEET6*, *ZmSUC2*), starch synthesis (*ZmAGPASE*, *ZmSS*), and sink strength (*ZmAINV1*) specifically in BR and SR, suggesting enhanced C turnover to sustain growth under N deficiency. In contrast, MN predominantly induced these genes in CR, LR, and PR, aligning with increased sugar accumulation and enzymatic activity in those root types (Liu et al. 2022).

### A coherent model of C–N coordination in maize roots under varying N availability

The integration of physiological, biochemical, and molecular responses reveals a coherent model of C–N coordination in maize roots under varying N availability. Under LN conditions, maize plants exhibit a strategic physiological adaptation by prioritizing root over shoot growth, resulting in a higher R/S ratio (Fig. S2A–C). This shift is achieved through precise architectural remodelling of the root system, in which the growth of BR, CR, PR, and SR is suppressed, while LR development is significantly promoted (Figs. S4–S6). This differential root-type growths lead to an extensive, branched network optimized for N exploration and uptake, contributing to increased total root length, number, volume, and surface area observed at the whole-root level (Figs. S2E–H) (Wu et al. 2024a). This physiological and architectural plasticity is driven by a coordinated biochemical and molecular program (Wang et al. 2025). Under LN, enhanced C allocation from shoots to roots results in higher levels of soluble sugars, sucrose, and starch in the whole root system (Fig. 1A–C and Fig. S7). This increased C supply provides essential energy and metabolic precursors for adaptive root growth. Notably, C is not distributed uniformly but is strategically allocated to specific root types based on N status (Fig. 5). Under LN, C reserves accumulate primarily in BR and PR, reinforcing structural integrity and deep rooting. In contrast, under MN conditions, C is preferentially directed to CR, LR, and SR to support active exploration and nutrient uptake (Fig. 5). The utilization and storage of C within each root type are tightly regulated by interconnected biochemical and molecular networks involving key enzymes and transporters (Wang and Ruan 2015; Wang et al. 2025).

At the molecular level, sucrose transporters (*ZmSWEET6*, *ZmSTP2*) and starch biosynthesis genes (*ZmAGPASE*, *ZmSS*) are broadly upregulated in whole roots under LN conditions (Fig. 7, 8, 9). However, their expression varies across root types (Figs. 7, 8, 9). Under LN, genes facilitating C turnover, such as *ZmSWEET6*, *ZmSUC2*, *ZmAGPASE*, *ZmSS*, and *ZmAINV1*, are predominantly induced in BR and SR, supporting metabolic activity in these structural and absorptive roots (Fig. 6, 7, 8, 9). Under MN, these genes are primarily expressed in CR, LR, and PR, aligning with observed root-type growth dynamics (Figs. 4, 6, 7, 8, 9). This differential molecular regulation ensures that the metabolic machinery for sucrose degradation and starch synthesis is active in root segments strategically favoured under each N condition. Enzymes such as SuSy and SUCs, supported by transporters, such as *ZmSTP2* and *ZmSWEET6*, facilitate sucrose breakdown, supplying energy and C skeletons for root cell division, elongation, and maintenance of the expanded root system. Starch accumulation serves as a critical energy reserve (Liang et al. 2023; Tian et al. 2024).

Temporal dynamics further underscore this coordination. Diurnal patterns show that LN-treated plants consistently maintain elevated sugar and starch levels throughout day and night cycles (Fig. 4), reflecting enhanced capacity for carbohydrate storage and utilization under N stress. Collectively, these findings support a coherent model in which N deficiency triggers a cascade of responses from the whole plant to the molecular level. Physiological adaptation involves resource reallocation and root architectural remodelling, particularly through differential growth of specific root types (Calleja-Cabrera et al. 2020; Ranjan et al. 2022). This is reinforced by enhanced C allocation and strategic partitioning across the root system (Fig. 5). The metabolic fate of this C is modulated by differential enzyme activities and the expression of genes involved in sucrose and starch metabolism, precisely regulated within each root type to match its functional role in the adaptive root architecture (Figs. 6, 7, 8, 9). This multi-layered, root-type-specific coordination of C and N metabolism enables maize to optimize root plasticity and improve NUE, which is essential for survival and productivity under N-limited conditions (Amoah and Kaiser 2025). These insights deepen our understanding of N-induced metabolic and molecular adaptations in maize roots, offering a valuable framework for crop improvement and sustainable agriculture. Ultimately, this model can inform N management strategies aimed at optimizing root function and enhancing resilience in low-input farming systems.

## Conclusion

The study examined the intricate relationship between N availability and C metabolism in maize roots, analysing responses at multiple levels: biomass accumulation, root morphology, and sugars and starch metabolism at both biochemical and transcriptional scales. Under LN conditions, maize seedlings exhibited significant adaptive responses. In terms of biomass accumulation and phenotypic adaptation, shoot growth was inhibited, while root growth was promoted, resulting in an increased R/S ratio. This enhancement in root development, characterized by greater root length, number, volume, and surface area, represents a strategic shift in resource allocation to improve N acquisition under N-limited conditions. Notably, LN treatment suppressed the growth of BR, CR, PR, and SR, while promoting LR development. These architectural modifications enhance how maize N from the soil. Supporting these physiological adaptations, biochemical analyses revealed significant metabolic shifts. LN treatment induced higher sugar and starch levels in maize roots, indicating increased transport of photosynthetic assimilates to the roots to provide essential energy and carbon skeletons for root growth and adaptation. These dynamic changes were driven by enhanced activities of key enzymes involved in sugar and starch metabolism, including SPS, SuSy, AGPASE, and SS. Furthermore, transcriptional analysis demonstrated a coordinated upregulation of genes associated with sucrose metabolism (*ZmSPS1*, *ZmSuSy1*, *ZmAINV1*, *ZmSWEET6*, *ZmSUC2*, and *ZmSTP2*) and starch biosynthesis (*ZmAGPASE1* and *ZmSS1*) under LN conditions. This suggests an active molecular program facilitating sucrose transport (*ZmSTP2*, *ZmSWEET6*, *ZmSUC2*), root sink strength (*ZmSuSy1*, *ZmAINV1*), and C storage (*ZmAGPASE1*, *ZmSS1*). Notably, these responses varied across different root types, indicating an enhanced regulatory network that modulates sucrose allocation and metabolic pathways to optimize maize adaptation to LN conditions. Collectively, these findings at morphological, biochemical, and molecular levels illustrate how maize coordinates a cohesive response to N deficiency. Molecular signals trigger metabolic adjustments that provide essential resources for adaptive root architectural modifications, ultimately enhancing NUE under N-limited conditions. These insights hold valuable implications for optimizing fertilization strategies and improving crop performance. Future studies will consider field validation across diverse genotypes and explore the anatomical, transcriptomic, and proteomic response of maize rooots to varying N levels to better understand root development and plasticity under N-deficiency conditions.

## Electronic supplementary material

Below is the link to the electronic supplementary material.

Supplementary Material 1

Supplementary Figures

## Data Availability

Data is contained in the manuscript.
