# Supplementary material for: Nitrogen deficiency identifies carbon metabolism pathways and root adaptation in maize
Source: Physiol Mol Biol Plants. 2025 Aug 6;31(7):1089–103. doi: 10.1007/s12298-025-01631-0 (PMC12394107; doi:10.1007/s12298-025-01631-0)
Supplement: Supplementary file 1 — Supplementary Material 1 [file 12298_2025_1631_MOESM1_ESM.docx]

**Table S1:** List of genes studied

| Name | NCBI LOCs |
| --- | --- |
| *ZmSuSy*  *ZmSPS*  *ZmSUC*  *ZmAGPase*  *ZmSTP*  *ZmSWEET*  *ZmVINV*  *ZmActin*  *ZmUBQc*  *ZmSS* | LOC542247  LOC542711  LOC541615  LOC542737  LOC100273083  LOC100282708  LOC542324  LOC100282267  LOC541665  LOC541669 |

**Table S2:** One-way analysis of variance (ANOVA) for indicators measured in roots of maize plants under different nitrogen conditions

| Traits/(plants) | Sources of variations | | | | | |
| --- | --- | --- | --- | --- | --- | --- |
|  |  | **Treatment (df=3)** | | | | |
|  | **Whole root** | **Brace root** | **Crown root** | **Lateral root** | **Primary root** | **Seminal root** |
| Root length  Root tip number  Root volume  Root surface area  Shoot biomass  Root biomass  Root:shoot ratio  Nitrogen  Soluble sugar  Sucrose  Starch  SPS activity  SuSy activity  AGPASE activity  Starch synthase  *ZmSPS*  *ZmSuSy*  *ZmAGPASE*  *ZmSS*  *ZmSWEET6*  *ZmSUC2*  *ZmSTP2*  *ZmAINV* | 0.0021**  0.0034**  0.0225*  <0.0001****  0.0028**  <0.0001****  <0.0001***  <0.0001***  <0.0001***  <0.0001***  <0.0001***  0.0026**  0.006**  <0.0001****  0.0011**  0.0005***  0.0003***  0.0002***  0.0019**  0.0003***  0.0006***  <0.0001****  <0.0001**** | NA  NA  NA  NA  NA  <0.0001****  NA  NA  0.0356*  0.0038**  0.0051**  0.0001***  0.0029**  0.0007***  0.0107*  0.0002***  0.0029**  <0.0001****  0.0041**  0.0004***  0.0013**  0.0002***  0.0035** | 0.0045**  0.0011**  0.0022**  0.0001***  NA  <0.0001****  NA  NA  0.0001***  0.0001***  0.0198*  0.0017**  0.0006***  0.0002***  0.0016**  <0.0001****  0.0006***  0.0002***  0.0058**  0.0003***  0.0005***  0.0001***  <0.0001**** | <0.0001****  0.0006***  0.0023**  <0.0001****  NA  0.0009***  NA  NA  0.0001***  <0.0001****  <0.0001****  0.0102*  0.1037ns  0.0095**  0.0069**  0.0156*  0.0035**  0.0443*  <0.0001****  0.002**  0.0129*  0.0008***  <0.0001**** | <0.0001****  0.0025**  0.0002***  <0.0001****  NA  0.1955ns  NA  NA  <0.0001****  0.0052**  <0.0001****  <0.0001****  <0.0001****  0.0046**  0.0001***  <0.0001****  <0.0001****  <0.0001****  <0.0001****  <0.0001****  <0.0001****  0.0026**  <0.0001**** | 0.0001***  <0.0001****  0.0004***  <0.0001****  NA  <0.0001****  NA  NA  0.004**  0.0105*  0.0006***  0.0002***  0.001**  <0.0001****  0.0013**  0.0138*  0.0019**  0.0095**  0.0004***  0.0459*  0.0012**  0.0011**  0.0028** |

SPS/*ZmSPS* (sucrose phosphate synthase), SuSy/*ZmSuSy* (sucrose synthase), and AGPASE/*ZmAGPASE1* (ADP-glucose pyrophosphorylase). The symbols *, **, ***, and **** indicate significant differences at probability levels of 0.5, 0.1, 0.001, and 0.0001, respectively. NA, ns, and df denote not applicable, non-significant, and degree of freedom, respectively.

**Table S3:** Sugars and starch metabolism enzymes activity in maize root types under different N forms

| Traits/(plants) | LN (1 mM NO_3_^-^) | Treatments  MN (2 mM NO_3_^-^) | HN (1 mM NO_3_^-^) | LA (10 mM NH_4_^+^) |
| --- | --- | --- | --- | --- |
|  | **SPS activity (µmol g^-1^ FW)** | | | |
| Brace root  Crown root  Lateral root  Primary root  Seminal root | 8.97 ± 0.41a  3.66 ± 0.12b  5.52 ± 0.30b  6.53 ± 0.66b  4.94 ± 0.08b | 3.45 ± 0.17d  5.89 ± 0.67a  6.37 ± 0.26a  12.11 ± 0.45a  7.87 ± 0.63a | 5.62 ± 0.51c  2.77 ± 0.26c  4.92 ± 0.04c  2.89 ± 0.09c  4.56 ± 0.06c | 6.80 ± 0.27b  2.25 ± 0.16d  4.44 ± 0.34d  3.13 ± 0.47c  3.38 ± 0.15c |
|  | **SuSy activity (µmol g^-1^ FW)** | | |  |
| Brace root  Crown root  Lateral root  Primary root  Seminal root | 11.36 ± 0.41a  3.57 ± 0.36c  7.11 ± 0.72b  8.84 ± 0.58b  6.98 ± 0.42b | 4.89 ± 0.22c  7.95 ± 0.74a  8.40 ± 0.35a  17.03 ± 0.59a  7.92 ± 0.48a | 8.94 ± 0.72b  4.879 ± 0.21b  6.23 ± 0.68c  3.77 ± 0.10c  6.00 ± 0.06c | 8.05 ± 1.06b  3.04 ± 0.22d  5.88 ± 0.44d  4.11 ± 0.62d  4.36 ± 0.15d |
|  | **AGPASE activity (µmol g^-1^ FW)** | | |  |
| Brace root  Crown root  Lateral root  Primary root  Seminal root | 6.35 ± 0.29a  2.75 ± 0.22b  5.53 ± 0.24b  6.45 ± 0.23a  3.95 ± 0.37b | 2.81 ± 0.23c  7.62 ± 0.68a  6.60 ± 0.30a  4.97 ± 0.52b  9.56 ± 0.43a | 3.25 ± 0.48b  2.77 ± 0.32b  3.97 ± 0.50c  3.97 ± 0.07c  2.41 ± 0.07c | 4.62 ± 0.25b  1.87 ± 0.42c  4.575 ± 0.33c  3.92 ± 0.32c  2.47 ± 0.48c |
|  | **SS activity (µmol g^-1^ FW)** | | |  |
| Brace root  Crown root  Lateral root  Primary root  Seminal root | 7.73 ± 0.17a  3.59 ± 0.45b  6.58 ± 0.29b  10.87 ± 0.40a  5.18 ± 0.25b | 4.50 ± 0.39c  6.59 ± 0.56a  8.01 ± 0.66a  7.30 ± 0.98b  6.49 ± 0.26a | 5.92 ± 0.52b  4.00 ± 0.05b  4.79 ± 0.45c  3.47 ± 0.15d  4.24 ± 0.22d | 6.45 ± 0.52b  2.93 ± 0.17c  5.02 ± 0.23c  3.69 ± 0.25c  4.84 ± 0.04c |

SPS (sucrose phosphate synthase), SuSy (sucrose synthase), AGPASE (ADP-glucose pyrophosphorylase), SS (sucrose synthase), LN (low nitrate), MN (medium nitrate), HN (high nitrate), LA (low ammonium nutrition), and FW (fresh weight of samples). The data represent the mean (±SE) of six independent plants (n=6). Different letters attached to the standard error indicate significant differences among treatments at a probability level of 0.05, as determined by one-way analysis of variance (ANOVA) followed by post-hoc comparisons.
