# Supplementary material for: Nitrogen deficiency identifies carbon metabolism pathways and root adaptation in maize
Source: Physiol Mol Biol Plants. 2025 Aug 6;31(7):1089–103. doi: 10.1007/s12298-025-01631-0 (PMC12394107; doi:10.1007/s12298-025-01631-0)
Supplement: Supplementary file 2 — Supplementary Figures [file 12298_2025_1631_MOESM2_ESM.docx]

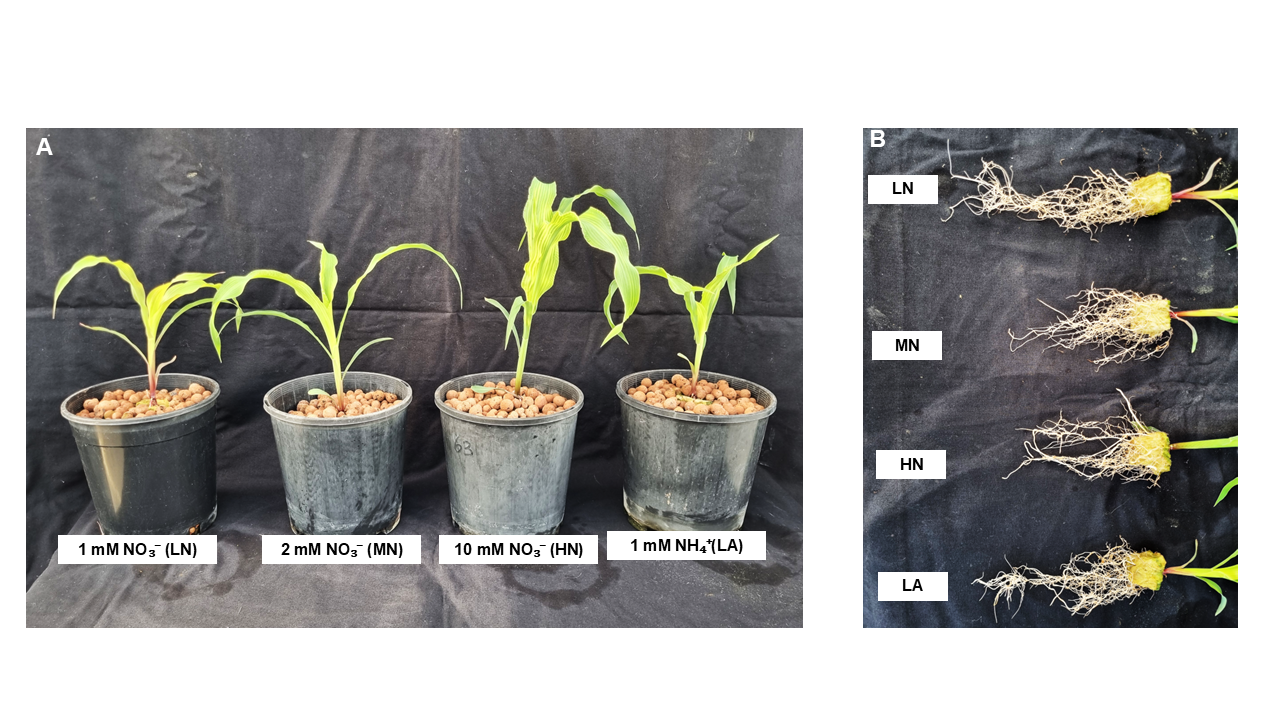


**Fig. S1.** Representative photos of shoot (A) and root (B) phenotypic response of maize seedlings to different N treatment conditions. LN: low nitrogen (N deficiency), MN: moderate nitrogen, HN: high nitrogen, and LA: low ammonium treatment.


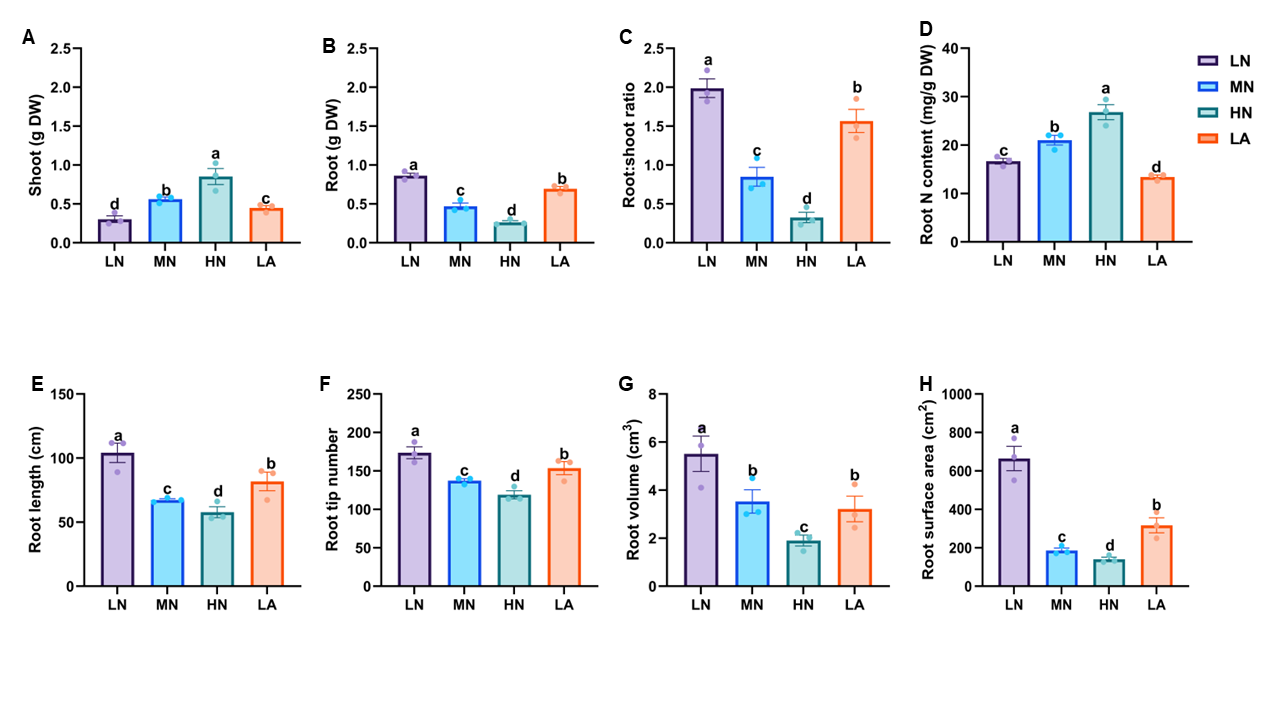


**Fig. S2.** Effects of different nitrogen (N) treatments on (A) shoot biomass, (B) whole-root biomass, (C) whole-root-to-shoot ratio, (D) whole-root nitrogen content, (E) whole-root length, (F) whole-root tip number, (G) whole-root volume, and (H) whole-root surface area in maize inbred line TX-40J. Data represents mean ± standard error (SE) of six independent plants (n = 6). Different letters on error bars indicate statistically significant differences at *P* ≤ 0.05. DW dry weight, LN low nitrate, MN moderate nitrate, HN high nitrate and LA low ammonium.


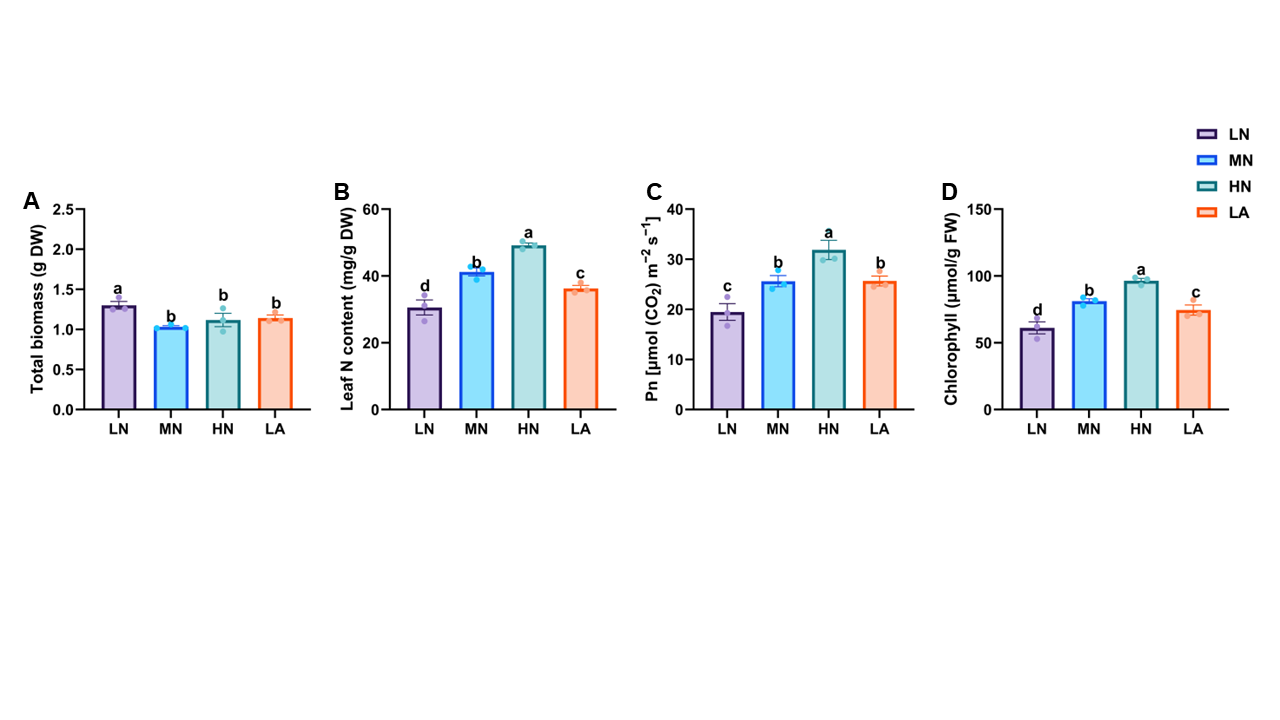


**Fig. S3.** Total biomass (A), leaf nitrogen content (B), leaf net photosynthetic rate (C) and leaf chlorophyll content (D) of maize seedlings grown under different nitrogen (N) treatments. Data represents the mean ± SEM (n = 6). Statistical significance was determined using Tukey's multiple range test (P< 0.05), with different letters indicating significant differences between treatments. DW denotes dry weights; LN: low nitrogen (N deficiency), MN: moderate nitrogen, HN: high nitrogen, and LA: low ammonium treatment


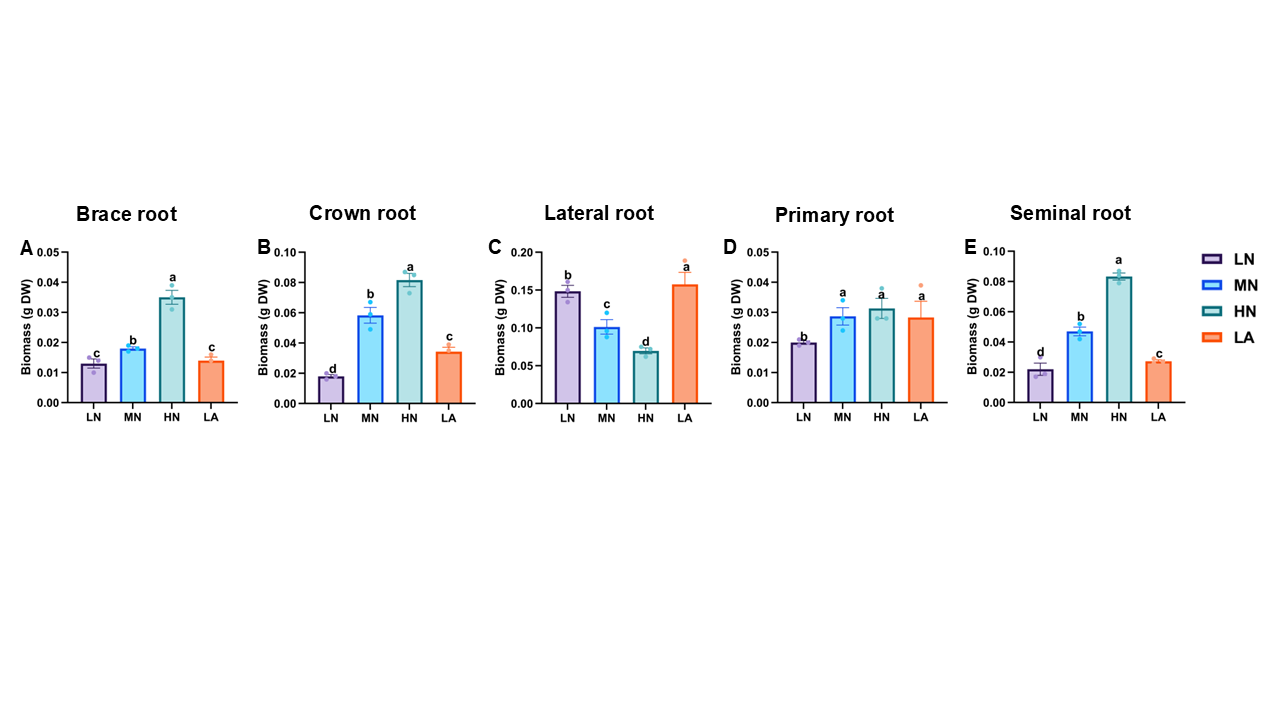


**Fig. S4:** Effect of different N forms on root biomass accumulation. Brace (A), crown (B), lateral (C), primary (D) and seminal (E) root biomass after 30 days of seedling transfer. Data are presented as the mean ± SEM (n = 6). Statistical significance was determined using Tukey's multiple range test (P< 0.05), with different letters indicating significant differences between treatments. DW denotes dry weights; LN: low nitrogen (N deficiency), MN: moderate nitrogen, HN: high nitrogen, and LA: low ammonium treatment.


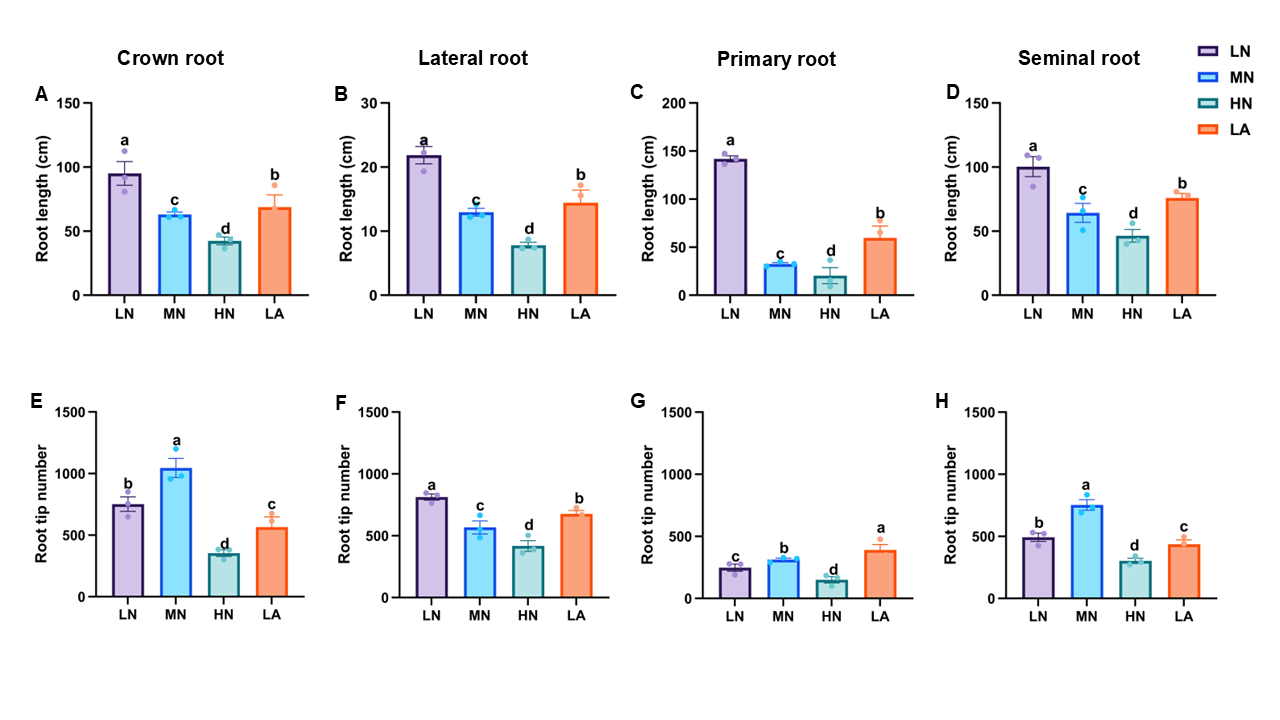


**Fig. S5:** Effect of different N forms on morphological response of different root types. Crown (A), lateral (B), primary (C) and seminal (D) root length (cm) and crown (A), lateral (B), primary (C) and seminal (D) root tip numbers. Data are presented as mean ± SEM (n = 6). Statistical significance was determined using Tukey's multiple range test (P< 0.05), with different letters indicating significant differences between treatments. LN: low nitrogen (N deficiency), MN: moderate nitrogen, HN: high nitrogen, and LA: low ammonium treatment.


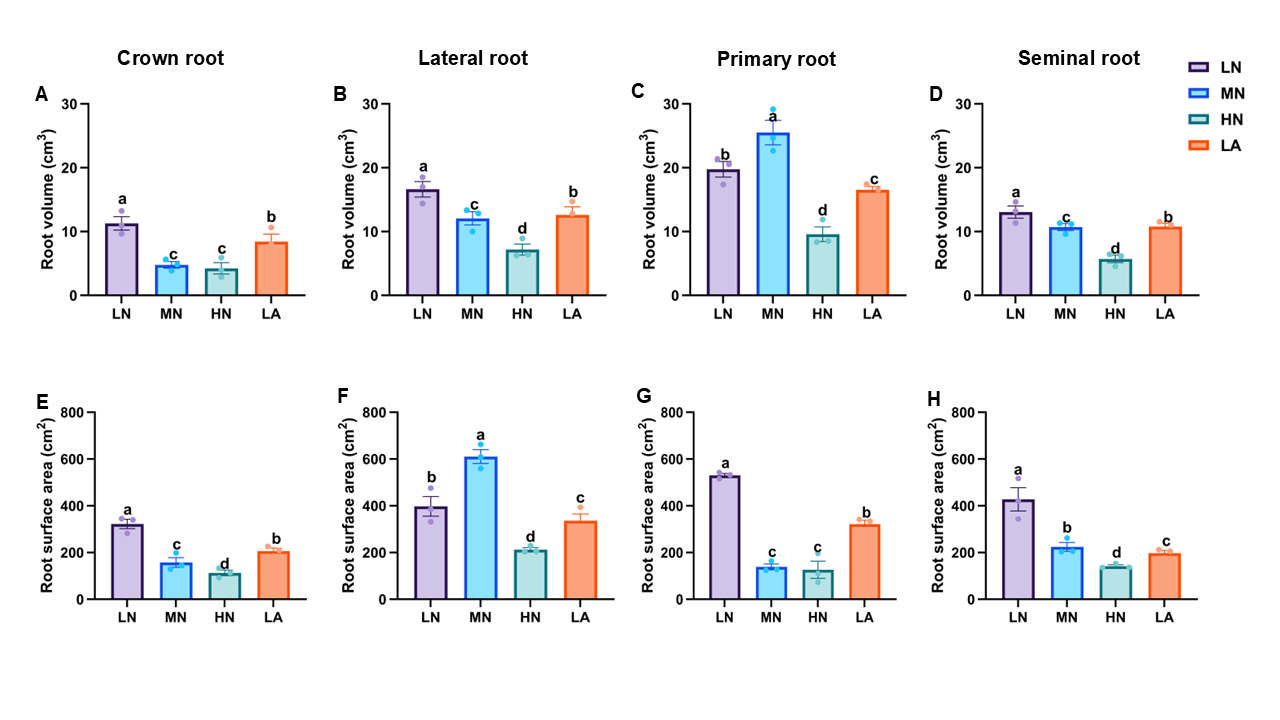


**Fig. S6:** Effect of different N forms on morphological response of different root types. Crown (A), lateral (B), primary (C) and seminal (D) root length and Crown (A), lateral (B), primary (C) and seminal (D) root surface area (cm^-2^). Data are presented as mean ± SEM (n = 6). Statistical significance was determined using Tukey's multiple range test (P< 0.05), with different letters indicating significant differences between treatments. LN: low nitrogen (LN), MN: moderate nitrogen, HN: high nitrogen, and LA: low ammonium treatment.


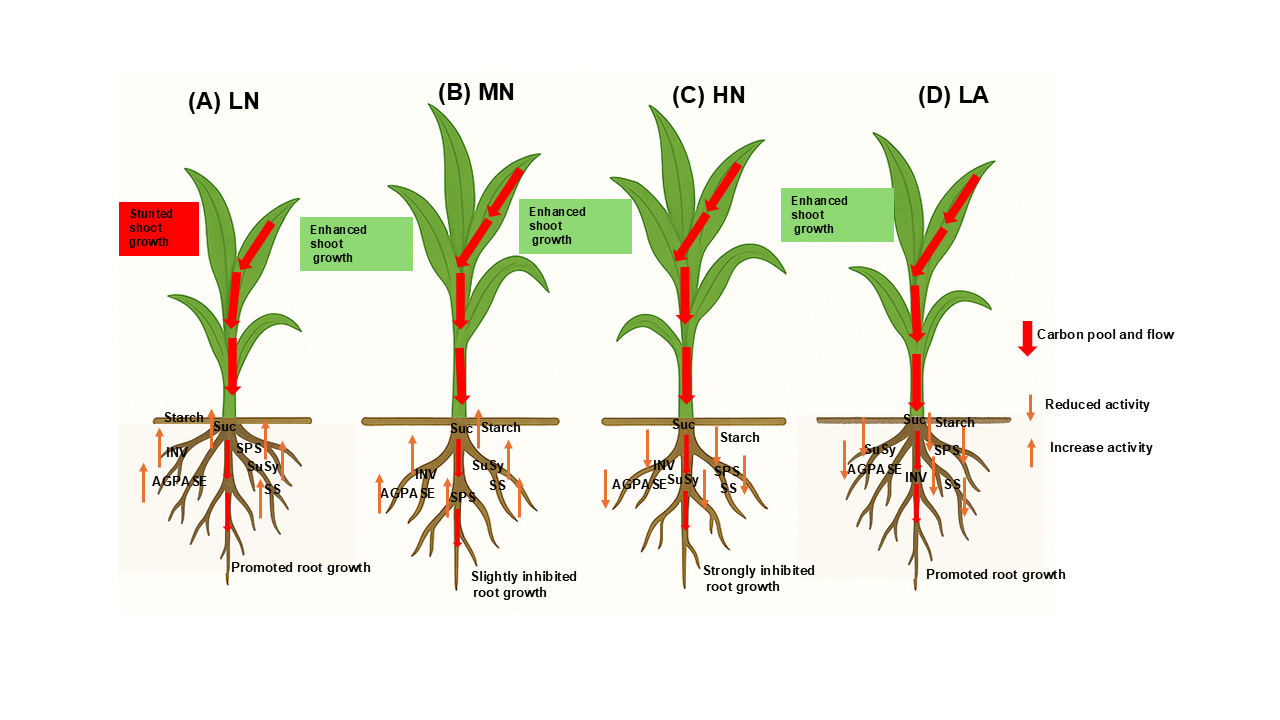


**Fig. S7:** Schematic diagram summarizing the changes occurring in maize due to different N treatments. Shoot and root growth, as well as carbon partitioning, varied markedly across treatments. Low nitrate (LN) reduced shoot growth while promoting root development, accompanied by diminished SPS activity and starch accumulation. Medium nitrate (MN) and high nitrate (HN) enhanced shoot growth with elevated SPS, SuSy, and AGPASE activities, though root growth was slightly or strongly inhibited, respectively. Low ammonium (LA) stimulated both shoot and root growth, suggesting enhanced coordination of sucrose metabolism and carbon flow, with increased INV, SuSy, AGPASE, and SPS activities. Abbreviations: Suc – sucrose, INV – invertase, SuSy – sucrose synthase, SS – starch synthase, AGPASE – ADP-glucose pyrophosphorylase, SPS – sucrose phosphate synthase, LN – low nitrate, MN – medium nitrate, HN – high nitrate, LA – low ammonium treatment. Suc: sucrose, INV: invertase, SuSy: sucrose synthase, SS: starch synthase, AGPASE: ADP-glucose pyrophosphorylase, SPS: sucrose phosphate synthase, LN: low nitrate, MN: medium nitrate, HN: high nitrate and LA: low ammonium treatment.


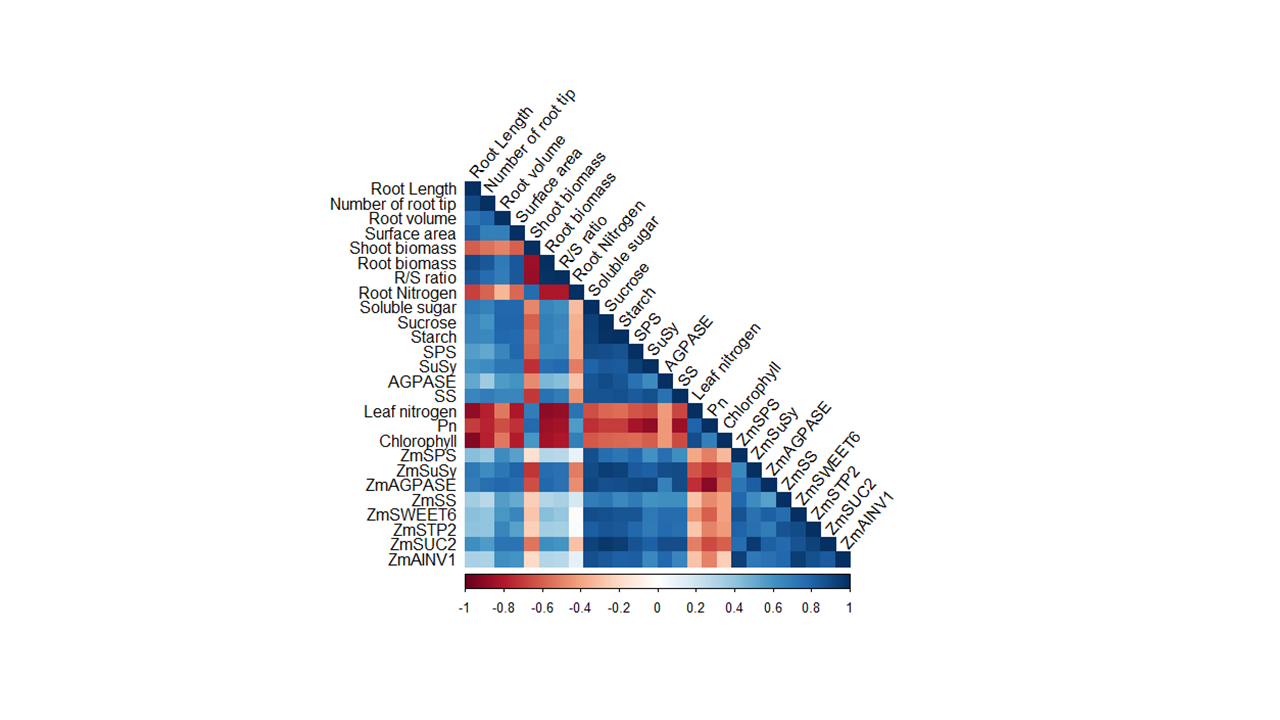


**Fig. S8.** Pearson’s correlation plot between physio-biochemical and molecular indicators under different nitrogen forms in maize roots. The plot was constructed using mean values of samples. SuSy/ZmSuSy: sucrose synthase, , ZmAINV1: acid/alkaline invertase, SPS/ZmSPS1: sucrose phosphate synthase, SS/ZmSS: starch synthase, AGPase/ZmAGPase1, ADP-glucose pyrophosphorylase , Pn: net photosynthetic rate, correlations are represented by colour gradients, ranging from blue (positive correlation) to red (negative correlation).
